# Supplementary material for: Molecular mechanism of BMP signal control by Twisted gastrulation
Source: Nat Commun. 2024 Jun 11;15:4976. doi: 10.1038/s41467-024-49065-8 (PMC11167000; doi:10.1038/s41467-024-49065-8)
Supplement: Supplementary file 1 — Supplementary Information [file 41467_2024_49065_MOESM1_ESM.pdf]

SUPPLEMENTARY INFORMATION

Molecular Mechanism of BMP Signal Control by Twisted Gastrulation

Tomas Malinauskas [1, *, **], Gareth Moore [2, *], Amalie F. Rudolf [1, *], Holly Eggington [3, 4], Hayley L. Belnoue-Davis [3, 4], Kamel El Omari [5], Samuel C. Griffiths [1, 6], Rachel E. Woolley [1, 7], Ramona Duman [5], Armin Wagner [5], Simon J. Leedham [3, 4], Clair Baldock [8], Hilary L. Ashe [2, **], Christian Siebold [1, **]

[1] *Division of Structural Biology, Wellcome Centre for Human Genetics, University of Oxford, Oxford OX3 7BN, UK.*

[2] *Faculty of Biology, Medicine and Health, University of Manchester, Manchester M13 9PT, UK.*

[3] *Intestinal Stem Cell Biology Lab, Wellcome Centre Human Genetics, University of Oxford, Oxford OX3 7BN, UK.*

[4] *Translational Gastroenterology Unit, John Radcliffe Hospital, University of Oxford, and Oxford National Institute for Health Research Biomedical Research Centre, Oxford, UK.*

[5] *Diamond Light Source, Harwell Science and Innovation Campus, Didcot OX11 0DE, UK.*

[6] *Current address: Evotec (UK) Ltd., 90 Innovation Drive, Milton Park, Abingdon OX14 4RZ, UK.*

[7] *Current address: Etcembly Ltd., Atlas Building, Harwell Campus, OX11 0QX, UK.*

[8] *Wellcome Centre for Cell-Matrix Research, Faculty of Biology, Medicine and Health, Manchester Academic Health Science Centre, University of Manchester, Manchester M13 9PT, UK.*

* These authors contributed equally and are listed in alphabetical order.

** These are corresponding authors: tomas@strubi.ox.ac.uk (T.M.), hilary.ashe@manchester.ac.uk (H.L.A.), christian@strubi.ox.ac.uk (C.S.).

SUPPLEMENTARY DISCUSSION

Modular architecture of TWSG1

The structure of TWSG1 reveals a modular architecture, comprising an α-helical N-terminal domain (NTD, Cys26–Arg80) connected by an extended 18 Å-long linker to a mixed-α/β C-terminal domain (CTD, Pro87–Phe223). The observed dimer is located on a crystallographic two-fold axis (**Fig. 1C**) resulting in a domain-swapped architecture with the NTD of one protomer interacting with the CTD of the second protomer. The interface is formed by mixed charged and hydrophobic interactions and is evolutionarily conserved (**Supplementary Fig. 1A–1B**). These interactions are mainly mediated by two interfaces: (1) helical regions of NTD and CTD docked onto each other and (2) edge-to-edge interactions between two antiparallel β-strands (Ser91–Val93) of the CTDs. The interface area between two TWSG1 molecules is 1869 $Å^2$ and 2025 $Å^2$ (crystal form 1), and 1896 $Å^2$ (crystal form 2), similar to interfaces observed in other protein-protein complexes (~1000–3000 $Å^2$)[1].

Structure of the N-terminal domain (NTD) of TWSG1

Search of structural homologues among all previously determined structures in the Protein Data Bank (PDB) did not yield any hits highlighting TWSG1's unique structural folds and their arrangement. The NTD of TWSG1 folds into a compact bundle of three α-helices locked by 7 disulfides: Cys26-Cys73 (I), Cys31-Cys70 (II), Cys38-Cys62 (III), Cys44-Cys59 (IV), Cys46-Cys55 (V), Cys53-Cys56 (VI), and Cys71-Cys77 (VII), which is further stabilized by a minimal hydrophobic core where Val35 and Leu39 from the α1-helix dock onto Leu63 of the α2-helix and Val74 of the α3-helix (**Fig. 1D**). The side chain of Trp67 intercalates between α2- and α3-helices and the hydrophobic core is stabilized by the side chain of Met76 inserted into a pocket framed by disulfides IV–VI. To investigate which residues of the NTD might mediate evolutionarily conserved TWSG1 interactions with its binding partners, we aligned 150 amino acid sequences most similar to the NTD. Mapping evolutionary conservation on the solvent-exposed surface of the NTD reveals a mixture of evolutionarily divergent, hydrophilic regions (two loops connecting the α1–α2 and α2–α3 helices; the C-terminal tail) as well as evolutionarily conserved hydrophobic residues (Ala29–Leu30 and Leu39–Ile40), suggesting that they are of functional importance (**Fig. 1D**, **Supplementary Fig. 1A–B**).

Next, we compared the TWSG1 NTD to experimentally determined structures and AlphaFold-derived models to illuminate evolutionary ties between the NTD and other proteins[2]. No experimentally determined structures similar to the TWSG1 NTD were found. AlphaFold-derived models of vertebrate homologues showed similarity to the crystal structure. Human TWSG1 NTD is predicted to be similar to the NTD of both Tsg (sequence identity 52.8%; RMSD of 0.92 Å for 48 aligned Cα atoms) and Crossveinless (sequence identity 50.9%; RMSD of 0.81 Å for 48 aligned Cα atoms) from *Drosophila*, explaining why Tsg and Crossveinless might be functionally equivalent[3] (**Supplementary Fig. 1C–D**). The TWSG1 NTD-like domain is also present in four human parasites, although the function of these TWSG1 NTD mimics has not been studied (**Supplementary Fig. 1E–H**).

Structure of the C-terminal domain (CTD) of TWSG1

The CTD of TWSG1 is stabilized by 5 disulfide bonds (VIII–XII) and contains a sheet comprised of 5 antiparallel β-strands which constitute its core (**Fig. 1E**). One side of the sheet is shielded from the solvent by two α-helices (α4 and α5). The first α4-helix is in the loop connecting the two edge strands (β1 and β2) of the core sheet. Leu96 of the α4-preceeding linker binds to the hydrophobic region of the core sheet cantered around Tyr193. Similarly, the hydrophobic side chains of Leu103, Phe104 and Leu107 anchor the α4-helix almost perpendicularly to β-strands 2–3 and 5. The α5-helix lies almost parallel to the central β-strand 5 and is stapled to it by two disulfide bonds (Cys181-Cys201 (X) and Cys185-Cys203 (XI)). The opposite face of the core sheet is enclosed by a secondary structure-lacking stretch of residues (Gly205–Phe223).

Similarly to the TWSG1 NTD, there are no experimentally determined structures resembling the CTD. However, the structure of CTD is conserved across species, from flies, parasitic nematodes to humans, as suggested by AlphaFold (**Supplementary Fig. 1A–H**). A TWSG1 CTD-like domain is present in the protein Shrew, which is required for the maximal activation of Dpp (BMP/GDF homologue) signaling in *Drosophila*[4]. To understand which residues of the CTD could be responsible for potential protein-protein interactions, we mapped evolutionarily conserved residues onto the solvent accessible surface of this domain. This analysis reveals conserved regions that could be important for TWSG1 function and Dpp/BMP signaling

(**Supplementary Fig. 1B**). Taken together, the crystal structure of human TWSG1 reveals two previously unseen, evolutionarily conserved disulfide-rich domains and points towards several surface-exposed residues that could modulate the BMP/GDF/Dpp signaling.

SUPPLEMENTARY FIGURES

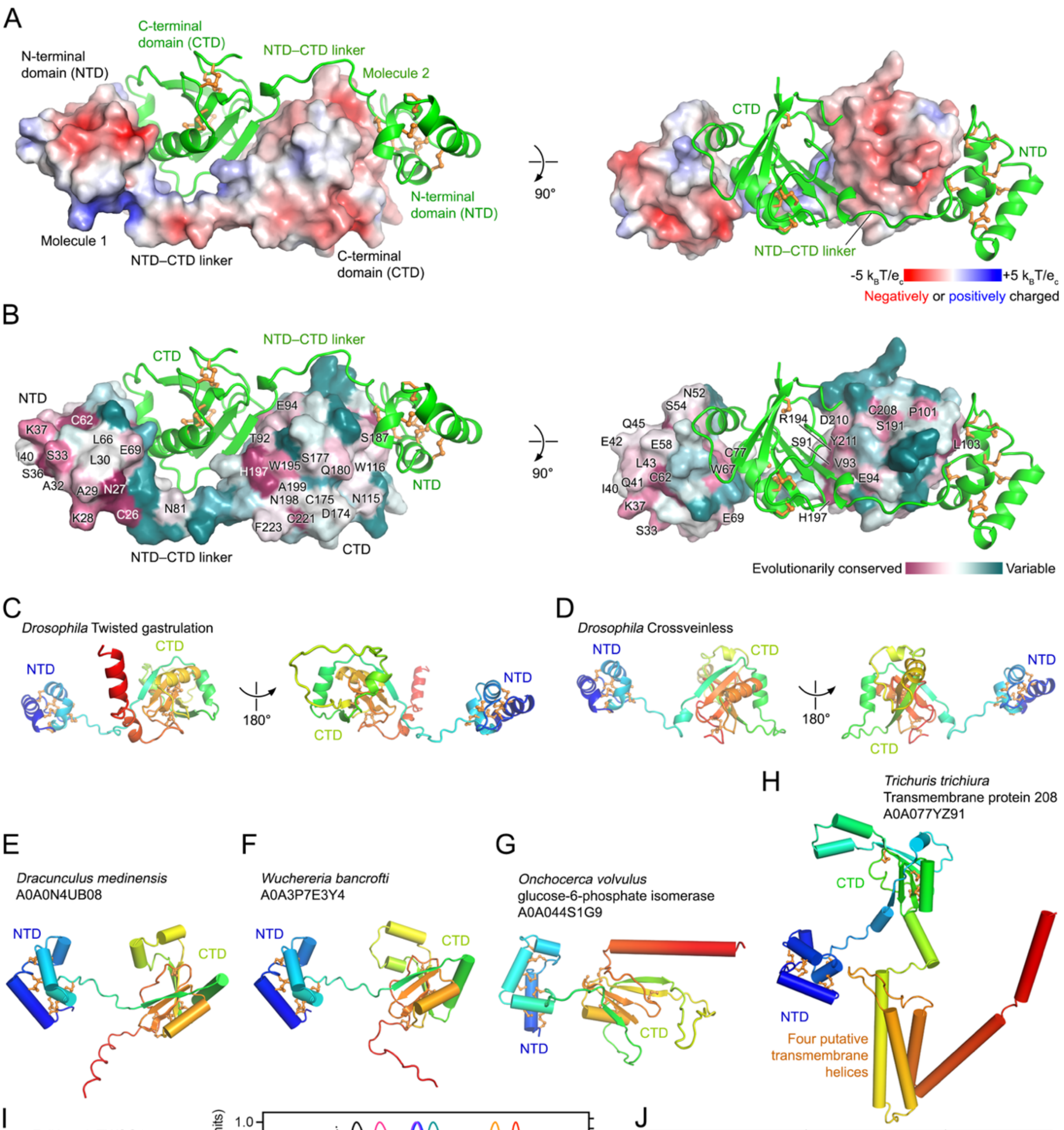

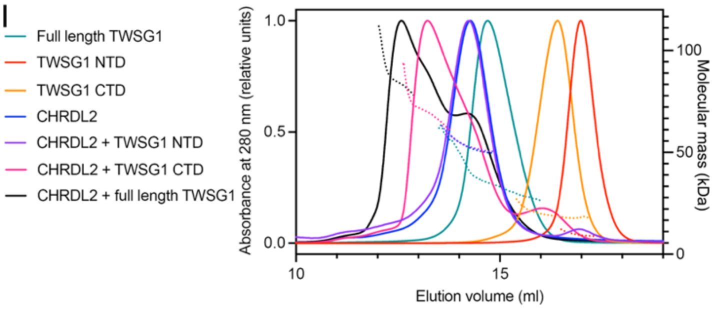

J

Protein or protein–protein complex (mol:mol)	Calculated molecular mass (protein + glycan), Da	Molecular mass observed using SEC-MALS, Da
Full length TWSG1	23540.66 + 5647.93	26332–61937
TWSG1 NTD	7494.71 + 3765.29	8830–12272
TWSG1 CTD	17124.18 + 1882.64	17568–27035
CHRDL2	46215.76 + 1882.64	49568–60538
CHRDL2 + TWSG1 NTD (1:1)	53710.47 + 5647.93	50399–63004
CHRDL2 + TWSG1 CTD (1:1)	63339.94 + 3765.29	60442–93423
CHRDL2 + full length TWSG1 (1:1)	69756.42 + 7530.57	77095–111861

Supplementary Fig. 1. Evolutionary ties of human TWSG1 to proteins from diverse organisms, and TWSG1 interactions with CHRDL2.

(**A** and **B**) Dimeric TWSG1 structure with electrostatic potential (**A**) and evolutionary conservation (**B**) mapped onto solvent accessible surface of one protomer. The second protomer is shown as green cartoon with disulfide bonds shown as orange spheres and sticks. Evolutionarily conserved residues are colored in purple and are numbered. (**C** and **D**) AlphaFold-derived models of Tsg (**C**) and Crossveinless (**D**) from *Drosophila*. Two views of each molecule differ by a 180° rotation around a vertical axis. Molecules are colored as rainbow (N-terminus, blue; C-terminus, red). (**E–H**) Proteins from four human parasites that contain domains similar to TWSG1 NTD and CTD. Evolutionary conserved disulfide bonds are colored in orange. (**I**) SEC-MALS analysis of three TWSG1 constructs (full length, NTD and CTD) and their interactions with CHRDL2. Traces of absorbance at 280 nm and calculated molecular masses are shown as continuous and dotted traces, respectively. CHRDL2 forms a complex with the full length TWSG1 (black trace) and TWSG1 CTD (pink) but not TWSG1 NTD (violet). (**J**) Summary of theoretical and SEC-MALS derived molecular masses of proteins analyzed using SEC-MALS (**I**). Calculated molar mass of one asparagine-linked glycan ($Man_9GlcNAc_2$) is 1882.64 Da.

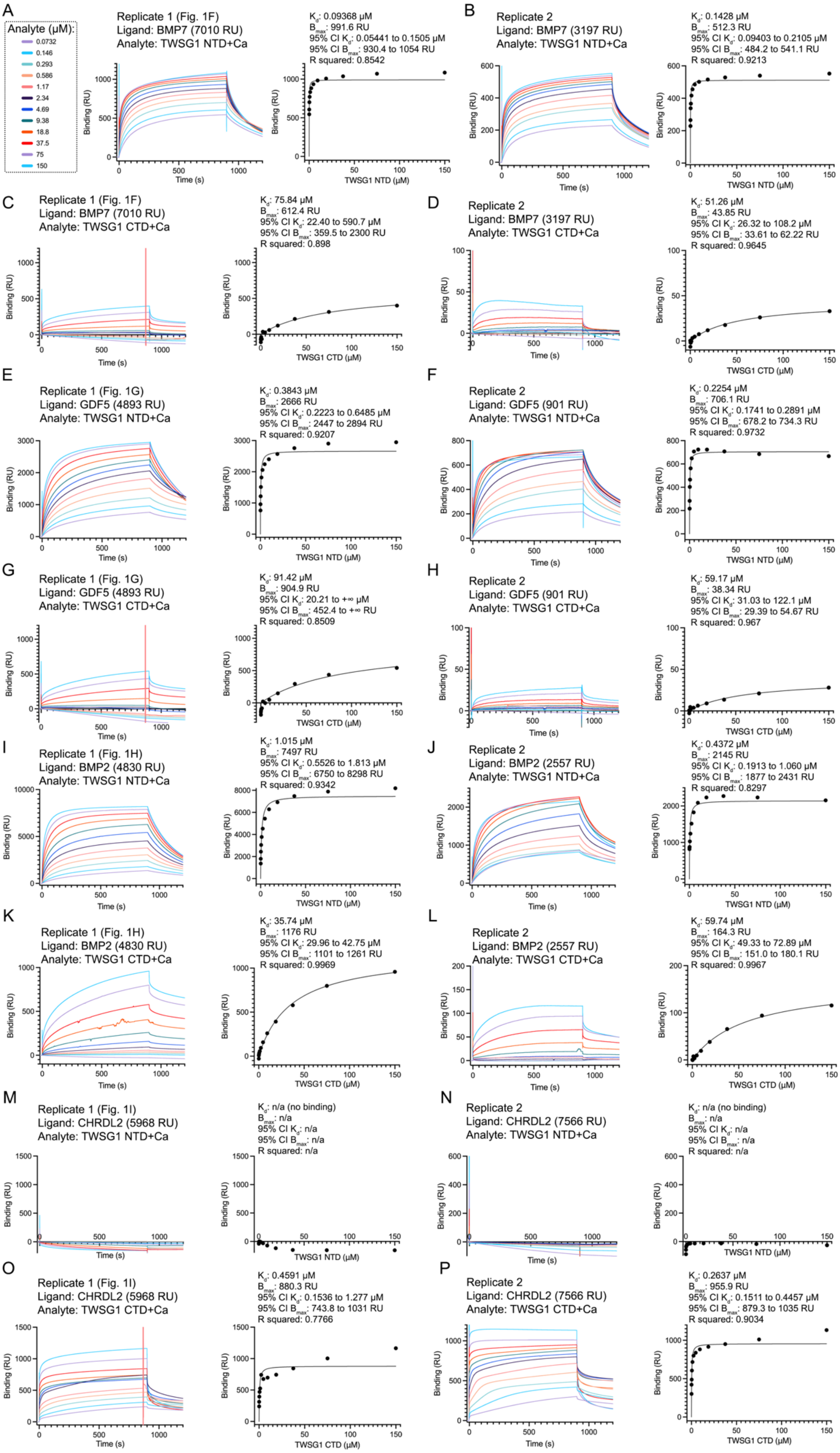
A
Analyte (µM):
0.0732
0.146
0.293
0.586
1.17
2.34
4.69
9.38
18.8
37.5
75
150
Replicate 1 (Fig. 1F)
Ligand: BMP7 (7010 RU)
Analyte: TWSG1 NTD+Ca
Kd: 0.09368 µM
Bmax: 991.6 RU
95% CI Kd: 0.05441 to 0.1505 µM
95% CI Bmax: 930.4 to 1054 RU
R squared: 0.8542
B
Replicate 2
Ligand: BMP7 (3197 RU)
Analyte: TWSG1 NTD+Ca
Kd: 0.1428 µM
Bmax: 512.3 RU
95% CI Kd: 0.09403 to 0.2105 µM
95% CI Bmax: 484.2 to 541.1 RU
R squared: 0.9213
C
Replicate 1 (Fig. 1F)
Ligand: BMP7 (7010 RU)
Analyte: TWSG1 CTD+Ca
Kd: 75.84 µM
Bmax: 612.4 RU
95% CI Kd: 22.40 to 590.7 µM
95% CI Bmax: 359.5 to 2300 RU
R squared: 0.898
D
Replicate 2
Ligand: BMP7 (3197 RU)
Analyte: TWSG1 CTD+Ca
Kd: 51.26 µM
Bmax: 43.85 RU
95% CI Kd: 26.32 to 108.2 µM
95% CI Bmax: 33.61 to 62.22 RU
R squared: 0.9645
E
Replicate 1 (Fig. 1G)
Ligand: GDF5 (4893 RU)
Analyte: TWSG1 NTD+Ca
Kd: 0.3843 µM
Bmax: 2666 RU
95% CI Kd: 0.2223 to 0.6485 µM
95% CI Bmax: 2447 to 2894 RU
R squared: 0.9207
F
Replicate 2
Ligand: GDF5 (901 RU)
Analyte: TWSG1 NTD+Ca
Kd: 0.2254 µM
Bmax: 706.1 RU
95% CI Kd: 0.1741 to 0.2891 µM
95% CI Bmax: 678.2 to 734.3 RU
R squared: 0.9732
G
Replicate 1 (Fig. 1G)
Ligand: GDF5 (4893 RU)
Analyte: TWSG1 CTD+Ca
Kd: 91.42 µM
Bmax: 904.9 RU
95% CI Kd: 20.21 to +∞ µM
95% CI Bmax: 452.4 to +∞ RU
R squared: 0.8509
H
Replicate 2
Ligand: GDF5 (901 RU)
Analyte: TWSG1 CTD+Ca
Kd: 59.17 µM
Bmax: 38.34 RU
95% CI Kd: 31.03 to 122.1 µM
95% CI Bmax: 29.39 to 54.67 RU
R squared: 0.967
I
Replicate 1 (Fig. 1H)
Ligand: BMP2 (4830 RU)
Analyte: TWSG1 NTD+Ca
Kd: 1.015 µM
Bmax: 7497 RU
95% CI Kd: 0.5526 to 1.813 µM
95% CI Bmax: 6750 to 8298 RU
R squared: 0.9342
J
Replicate 2
Ligand: BMP2 (2557 RU)
Analyte: TWSG1 NTD+Ca
Kd: 0.4372 µM
Bmax: 2145 RU
95% CI Kd: 0.1913 to 1.060 µM
95% CI Bmax: 1877 to 2431 RU
R squared: 0.8297
K
Replicate 1 (Fig. 1H)
Ligand: BMP2 (4830 RU)
Analyte: TWSG1 CTD+Ca
Kd: 35.74 µM
Bmax: 1176 RU
95% CI Kd: 29.96 to 42.75 µM
95% CI Bmax: 1101 to 1261 RU
R squared: 0.9969
L
Replicate 2
Ligand: BMP2 (2557 RU)
Analyte: TWSG1 CTD+Ca
Kd: 59.74 µM
Bmax: 164.3 RU
95% CI Kd: 49.33 to 72.89 µM
95% CI Bmax: 151.0 to 180.1 RU
R squared: 0.9967
M
Replicate 1 (Fig. 1I)
Ligand: CHRDL2 (5968 RU)
Analyte: TWSG1 NTD+Ca
Kd: n/a (no binding)
Bmax: n/a
95% CI Kd: n/a
95% CI Bmax: n/a
R squared: n/a
N
Replicate 2
Ligand: CHRDL2 (7566 RU)
Analyte: TWSG1 NTD+Ca
Kd: n/a (no binding)
Bmax: n/a
95% CI Kd: n/a
95% CI Bmax: n/a
R squared: n/a
O
Replicate 1 (Fig. 1I)
Ligand: CHRDL2 (5968 RU)
Analyte: TWSG1 CTD+Ca
Kd: 0.4591 µM
Bmax: 880.3 RU
95% CI Kd: 0.1536 to 1.277 µM
95% CI Bmax: 743.8 to 1031 RU
R squared: 0.7766
P
Replicate 2
Ligand: CHRDL2 (7566 RU)
Analyte: TWSG1 CTD+Ca
Kd: 0.2637 µM
Bmax: 955.9 RU
95% CI Kd: 0.1511 to 0.4457 µM
95% CI Bmax: 879.3 to 1035 RU
R squared: 0.9034
Binding (RU)
Time (s)
TWSG1 NTD (µM)
TWSG1 CTD (µM)

Supplementary Fig. 2. SPR-based equilibrium binding experiments between individual domains of TWSG1 and their binding partners (BMPs and CHRDL2).

(**A–D**) SPR-based equilibrium binding experiments showing TWSG1 NTD–BMP7 (**A** and **B**, performed in duplicate) and TWSG1 CTD–BMP7 (**C** and **D**, performed in duplicate) interactions. Both SPR sensorgrams and corresponding isotherms are shown.

(**E–H**) SPR-based equilibrium binding experiments showing TWSG1 NTD–GDF5 (**E** and **F**, performed in duplicate) and TWSG1 CTD–GDF5 (**G** and **H**, performed in duplicate) interactions. Both SPR sensorgrams and corresponding isotherms are shown.

(**I–L**) SPR-based equilibrium binding experiments showing TWSG1 NTD–BMP2 (**I** and **J**, performed in duplicate) and TWSG1 CTD–BMP2 (**K** and **L**, performed in duplicate) interactions. Both SPR sensorgrams and corresponding isotherms are shown.

(**M–P**) SPR-based equilibrium binding experiments between TWSG1 NTD–CHRDL2 (**M** and **N**, performed in duplicate) and TWSG1 CTD–BMP2 (**O** and **P**, performed in duplicate). TWSG1 NTD did not bind to CHRDL2. Both SPR sensorgrams and corresponding isotherms are shown. Twelve different concentrations of the analyte (TWSG1 NTD or CTD) ranging from 0.0732 µM to 150 µM were injected over SPR chip with BMP7, GDF5, BMP2 or CHRDL2 ligands. The analyte concentrations associated with each sensorgram are depicted in panel **A**. Experiments were performed in the presence of 2 mM $CaCl_2$. Amount (resonance units, RU) of immobilized ligand on the chip is indicated. K_d, equilibrium binding dissociation constant; B_{max}, maximum response at saturating concentration of analyte; 95% CI, 95% Confidence Intervals, both for K_d and B_{max} are indicated; RU, resonance units; n/a, not applicable.

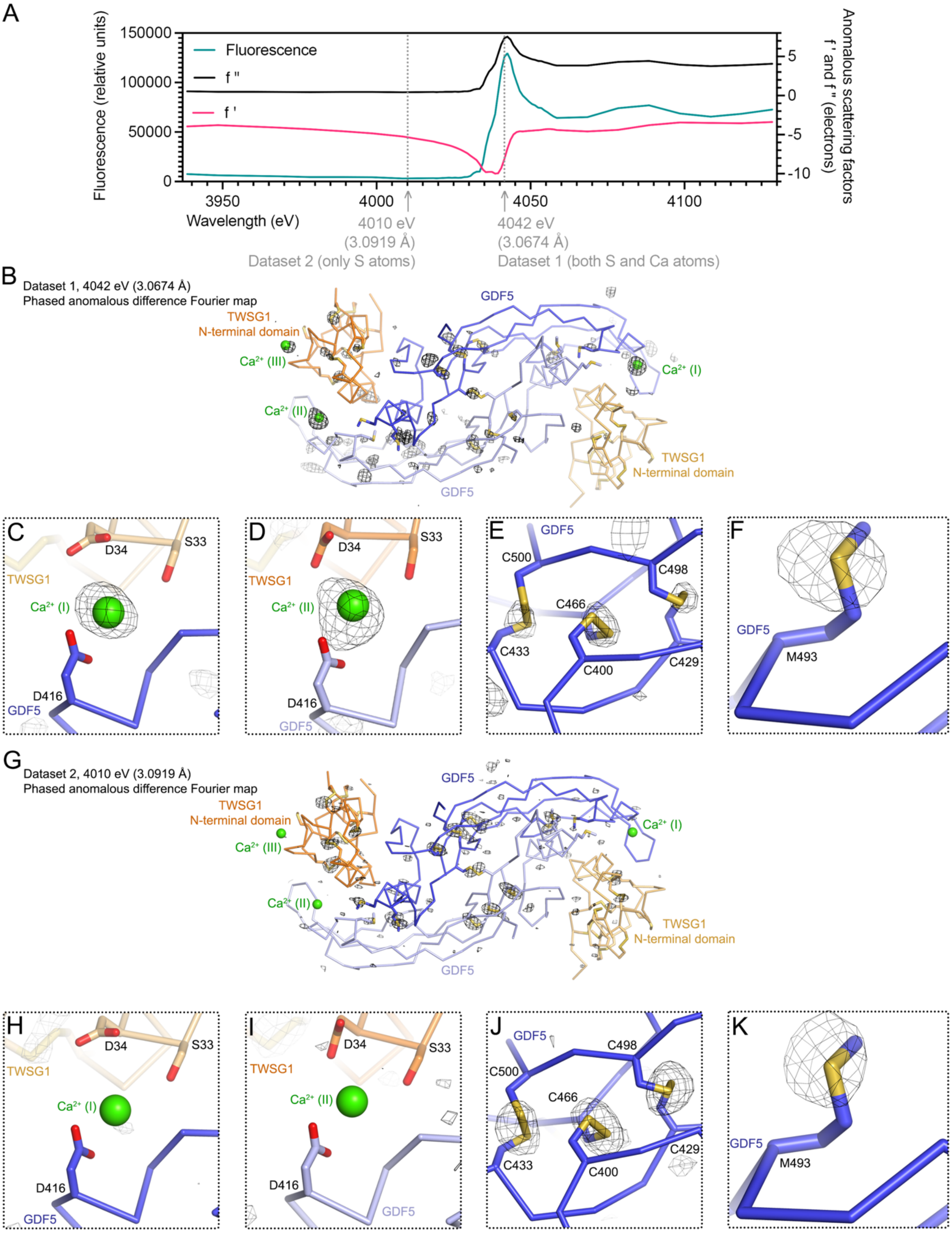

A
Fluorescence
f ''
f '
Fluorescence (relative units)
Anomalous scattering factors f ' and f '' (electrons)
Wavelength (eV)
4010 eV (3.0919 Å) Dataset 2 (only S atoms)
4042 eV (3.0674 Å) Dataset 1 (both S and Ca atoms)
B
Dataset 1, 4042 eV (3.0674 Å)
Phased anomalous difference Fourier map
TWSG1 N-terminal domain
GDF5
Ca²⁺ (I)
Ca²⁺ (II)
Ca²⁺ (III)
C
D
E
F
D34
S33
D416
C500
C498
C466
C433
C400
C429
M493
G
Dataset 2, 4010 eV (3.0919 Å)
Phased anomalous difference Fourier map
H
I
J
K

Supplementary Fig. 3. Analysis of the TWSG1–GDF5–calcium complex using the long-wavelength X-rays.

(**A**) Fluorescence scan of the TWSG1–GDF5–calcium crystal across the Ca K absorption edge (E = 4038.1 eV, λ = 3.0703 Å) carried out at the Diamond Light Source beamline I23. Anomalous scattering factors f′ (pink trace) and f′′ (black) derived from X-ray fluorescence spectra (aquamarine) are shown. Two X-ray diffraction data sets were collected using the same TWSG1–GDF5–calcium crystal at 4042 eV (dataset 1) and 4010 eV (dataset 2) to maximize anomalous X-ray scattering by calcium and sulphur (dataset 1), or by sulphur atoms only (dataset 2). (**B**) Crystal structure of the TWSG1–GDF5–calcium complex determined at 4042 eV (dataset 1). Two protomers of the dimeric GDF5 are shown as ribbons and colored in dark and light blue. Two TWSG1 NTDs are colored in dark and light orange. Three calcium ions are shown as green spheres. Phased anomalous difference Fourier map is shown as black mesh and is contoured at 3σ level in PyMOL. (**C–D**) Two calcium binding sites at the TWSG1–GDF5 interface and associated phased anomalous difference Fourier maps derived from dataset 1 (4042 eV). (**E–F**) Three disulfide bonds (**E**) and Met493 (**F**) of GDF5 plus associated phased anomalous difference Fourier maps calculated from dataset 1 (4042 eV). (**G**) Crystal structure of the TWSG1–GDF5–calcium complex determined at 4010 eV (dataset 2). Two protomers of the dimeric GDF5 are shown as ribbons and colored in dark and light blue. Two TWSG1 NTDs are colored in dark and light orange. Three calcium ions are shown as green spheres. Phased anomalous difference Fourier map is shown as black mesh and is contoured at 3σ level in PyMOL. (**H–I**) Two calcium binding sites at the TWSG1–GDF5 interface and associated phased anomalous difference Fourier maps derived from dataset 2 (4010 eV). Note the specific absence of anomalous scattering signal from calcium atoms as expected at this wavelength which is below the calcium K absorption edge. (**J–K**) Three disulfide bonds (**J**) and Met493 (**K**) of GDF5 plus associated phased anomalous difference Fourier maps derived from dataset 2 (4010 eV). Note anomalous scattering signal from sulphur atoms as expected at this wavelength, suggesting that the absence of anomalous scattering by calcium atoms (**H–I**) is due to the wavelength used (4010 eV) but not due to radiation damage.

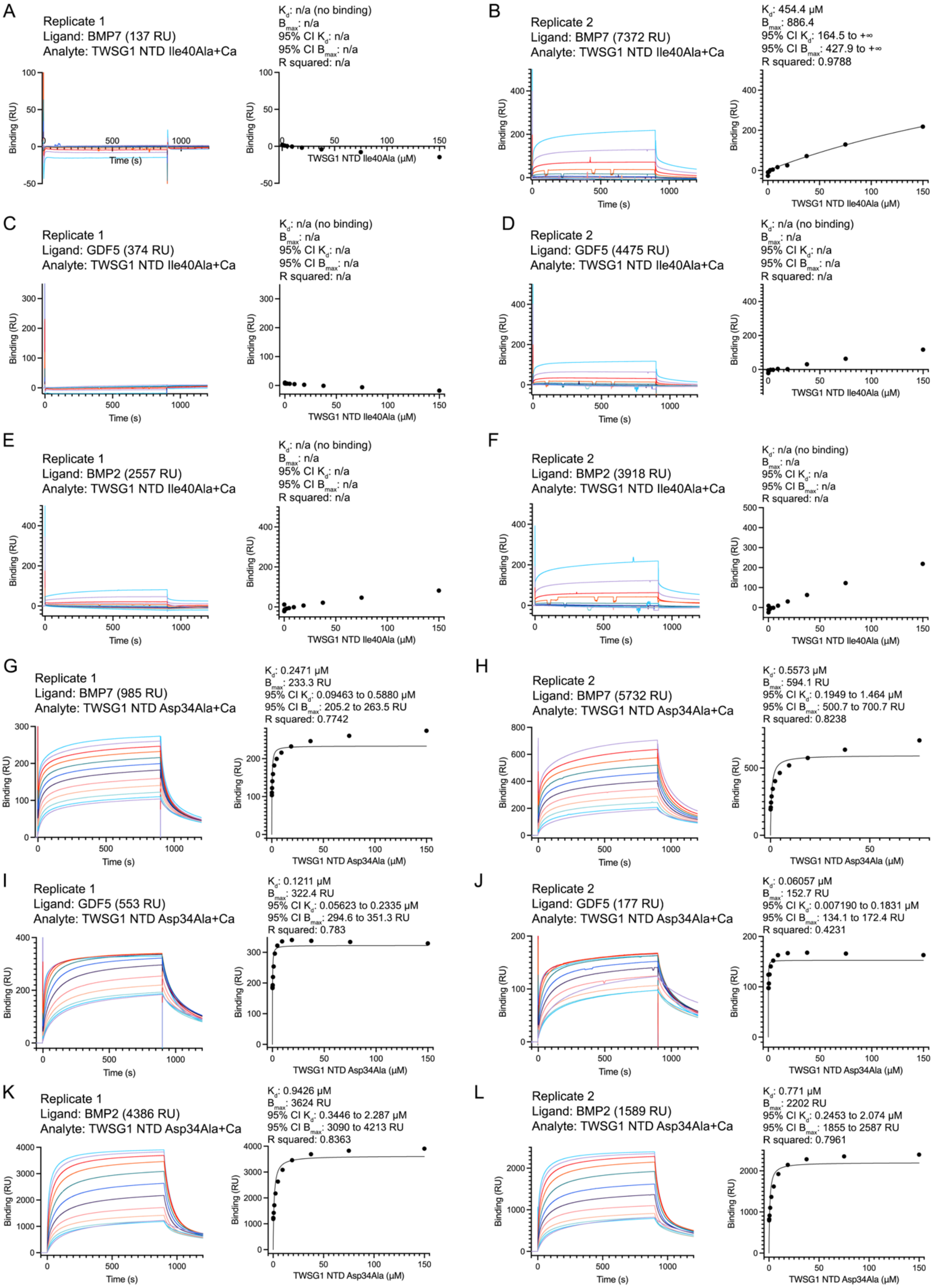
A
Replicate 1
Ligand: BMP7 (137 RU)
Analyte: TWSG1 NTD Ile40Ala+Ca
Kd: n/a (no binding)
Bmax: n/a
95% CI Kd: n/a
95% CI Bmax: n/a
R squared: n/a
B
Replicate 2
Ligand: BMP7 (7372 RU)
Analyte: TWSG1 NTD Ile40Ala+Ca
Kd: 454.4 μM
Bmax: 886.4
95% CI Kd: 164.5 to +∞
95% CI Bmax: 427.9 to +∞
R squared: 0.9788
C
Replicate 1
Ligand: GDF5 (374 RU)
Analyte: TWSG1 NTD Ile40Ala+Ca
Kd: n/a (no binding)
Bmax: n/a
95% CI Kd: n/a
95% CI Bmax: n/a
R squared: n/a
D
Replicate 2
Ligand: GDF5 (4475 RU)
Analyte: TWSG1 NTD Ile40Ala+Ca
Kd: n/a (no binding)
Bmax: n/a
95% CI Kd: n/a
95% CI Bmax: n/a
R squared: n/a
E
Replicate 1
Ligand: BMP2 (2557 RU)
Analyte: TWSG1 NTD Ile40Ala+Ca
Kd: n/a (no binding)
Bmax: n/a
95% CI Kd: n/a
95% CI Bmax: n/a
R squared: n/a
F
Replicate 2
Ligand: BMP2 (3918 RU)
Analyte: TWSG1 NTD Ile40Ala+Ca
Kd: n/a (no binding)
Bmax: n/a
95% CI Kd: n/a
95% CI Bmax: n/a
R squared: n/a
G
Replicate 1
Ligand: BMP7 (985 RU)
Analyte: TWSG1 NTD Asp34Ala+Ca
Kd: 0.2471 μM
Bmax: 233.3 RU
95% CI Kd: 0.09463 to 0.5880 μM
95% CI Bmax: 205.2 to 263.5 RU
R squared: 0.7742
H
Replicate 2
Ligand: BMP7 (5732 RU)
Analyte: TWSG1 NTD Asp34Ala+Ca
Kd: 0.5573 μM
Bmax: 594.1 RU
95% CI Kd: 0.1949 to 1.464 μM
95% CI Bmax: 500.7 to 700.7 RU
R squared: 0.8238
I
Replicate 1
Ligand: GDF5 (553 RU)
Analyte: TWSG1 NTD Asp34Ala+Ca
Kd: 0.1211 μM
Bmax: 322.4 RU
95% CI Kd: 0.05623 to 0.2335 μM
95% CI Bmax: 294.6 to 351.3 RU
R squared: 0.783
J
Replicate 2
Ligand: GDF5 (177 RU)
Analyte: TWSG1 NTD Asp34Ala+Ca
Kd: 0.06057 μM
Bmax: 152.7 RU
95% CI Kd: 0.007190 to 0.1831 μM
95% CI Bmax: 134.1 to 172.4 RU
R squared: 0.4231
K
Replicate 1
Ligand: BMP2 (4386 RU)
Analyte: TWSG1 NTD Asp34Ala+Ca
Kd: 0.9426 μM
Bmax: 3624 RU
95% CI Kd: 0.3446 to 2.287 μM
95% CI Bmax: 3090 to 4213 RU
R squared: 0.8363
L
Replicate 2
Ligand: BMP2 (1589 RU)
Analyte: TWSG1 NTD Asp34Ala+Ca
Kd: 0.771 μM
Bmax: 2202 RU
95% CI Kd: 0.2453 to 2.074 μM
95% CI Bmax: 1855 to 2587 RU
R squared: 0.7961
Binding (RU)
Time (s)
TWSG1 NTD Ile40Ala (μM)
TWSG1 NTD Asp34Ala (μM)

Supplementary Fig. 4. SPR-based equilibrium binding experiments between two variants of TWSG1 NTD (Ile40Ala or Asp34Ala) and three BMPs.

(**A–B**) SPR-based equilibrium binding experiments between TWSG1 NTD Ile40Ala and BMP7. Both SPR sensorgrams and corresponding isotherms are shown. Experiments were performed in duplicate.

(**C–D**) SPR-based equilibrium binding experiments between TWSG1 NTD Ile40Ala and GDF5. Both SPR sensorgrams and corresponding isotherms are shown. Experiments were performed in duplicate.

(**E–F**) SPR-based equilibrium binding experiments between TWSG1 NTD Ile40Ala and BMP2. Both SPR sensorgrams and corresponding isotherms are shown. Experiments were performed in duplicate.

(**G–H**) SPR-based equilibrium binding experiments between TWSG1 NTD Asp34Ala and BMP7. Both SPR sensorgrams and corresponding isotherms are shown. Experiments were performed in duplicate.

(**I–J**) SPR-based equilibrium binding experiments between TWSG1 NTD Asp34Ala and GDF5. Both SPR sensorgrams and corresponding isotherms are shown. Experiments were performed in duplicate.

(**K–L**) SPR-based equilibrium binding experiments between TWSG1 NTD Asp34Ala and BMP2. Both SPR sensorgrams and corresponding isotherms are shown. Experiments were performed in duplicate.

Twelve different concentrations of the analyte (TWSG1 NTD Ile40Ala or Asp34Ala) ranging from 0.0732 µM to 150 µM were injected over SPR chip with BMP7, GDF5, or BMP2 ligands. The analyte concentrations associated with each sensorgram are depicted in **Supplementary Fig. 2A**. Experiments were performed in the presence of 2 mM $CaCl_2$. The highest concentration of TWSG1 NTD Asp34Ala in the binding experiment with BMP7 (replicate 2) was 75 µM (**H**). Amount (RU) of immobilized ligand on the chip is indicated. K_d, equilibrium binding dissociation constant; B_{max}, maximum response at saturating concentration of analyte; 95% CI, 95% Confidence Intervals, both for K_d and B_{max}, are indicated; RU, resonance units; n/a, not applicable.

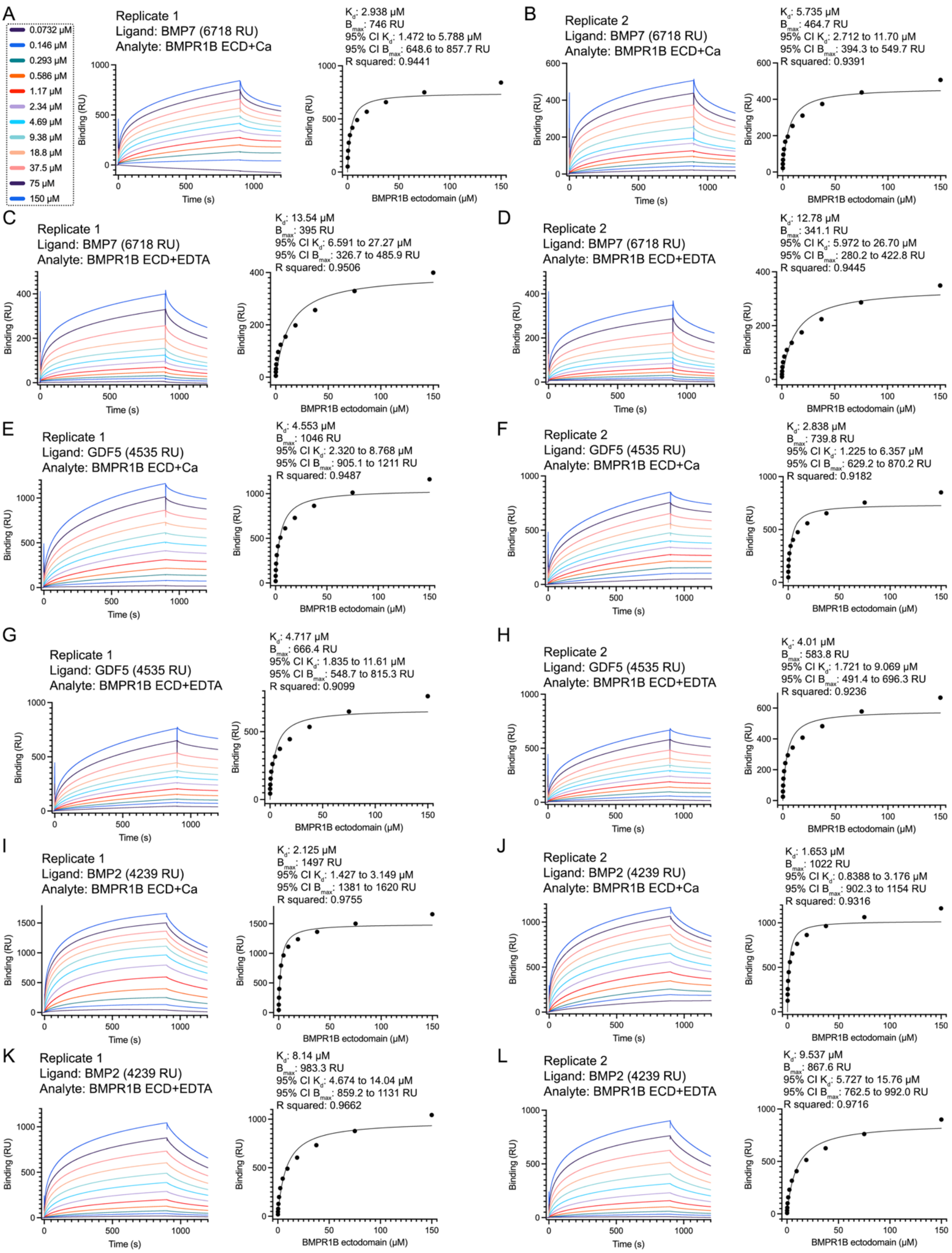

A
0.0732 μM
0.146 μM
0.293 μM
0.586 μM
1.17 μM
2.34 μM
4.69 μM
9.38 μM
18.8 μM
37.5 μM
75 μM
150 μM
Replicate 1
Ligand: BMP7 (6718 RU)
Analyte: BMPR1B ECD+Ca
Kd: 2.938 μM
Bmax: 746 RU
95% CI Kd: 1.472 to 5.788 μM
95% CI Bmax: 648.6 to 857.7 RU
R squared: 0.9441
B
Replicate 2
Ligand: BMP7 (6718 RU)
Analyte: BMPR1B ECD+Ca
Kd: 5.735 μM
Bmax: 464.7 RU
95% CI Kd: 2.712 to 11.70 μM
95% CI Bmax: 394.3 to 549.7 RU
R squared: 0.9391
C
Replicate 1
Ligand: BMP7 (6718 RU)
Analyte: BMPR1B ECD+EDTA
Kd: 13.54 μM
Bmax: 395 RU
95% CI Kd: 6.591 to 27.27 μM
95% CI Bmax: 326.7 to 485.9 RU
R squared: 0.9506
D
Replicate 2
Ligand: BMP7 (6718 RU)
Analyte: BMPR1B ECD+EDTA
Kd: 12.78 μM
Bmax: 341.1 RU
95% CI Kd: 5.972 to 26.70 μM
95% CI Bmax: 280.2 to 422.8 RU
R squared: 0.9445
E
Replicate 1
Ligand: GDF5 (4535 RU)
Analyte: BMPR1B ECD+Ca
Kd: 4.553 μM
Bmax: 1046 RU
95% CI Kd: 2.320 to 8.768 μM
95% CI Bmax: 905.1 to 1211 RU
R squared: 0.9487
F
Replicate 2
Ligand: GDF5 (4535 RU)
Analyte: BMPR1B ECD+Ca
Kd: 2.838 μM
Bmax: 739.8 RU
95% CI Kd: 1.225 to 6.357 μM
95% CI Bmax: 629.2 to 870.2 RU
R squared: 0.9182
G
Replicate 1
Ligand: GDF5 (4535 RU)
Analyte: BMPR1B ECD+EDTA
Kd: 4.717 μM
Bmax: 666.4 RU
95% CI Kd: 1.835 to 11.61 μM
95% CI Bmax: 548.7 to 815.3 RU
R squared: 0.9099
H
Replicate 2
Ligand: GDF5 (4535 RU)
Analyte: BMPR1B ECD+EDTA
Kd: 4.01 μM
Bmax: 583.8 RU
95% CI Kd: 1.721 to 9.069 μM
95% CI Bmax: 491.4 to 696.3 RU
R squared: 0.9236
I
Replicate 1
Ligand: BMP2 (4239 RU)
Analyte: BMPR1B ECD+Ca
Kd: 2.125 μM
Bmax: 1497 RU
95% CI Kd: 1.427 to 3.149 μM
95% CI Bmax: 1381 to 1620 RU
R squared: 0.9755
J
Replicate 2
Ligand: BMP2 (4239 RU)
Analyte: BMPR1B ECD+Ca
Kd: 1.653 μM
Bmax: 1022 RU
95% CI Kd: 0.8388 to 3.176 μM
95% CI Bmax: 902.3 to 1154 RU
R squared: 0.9316
K
Replicate 1
Ligand: BMP2 (4239 RU)
Analyte: BMPR1B ECD+EDTA
Kd: 8.14 μM
Bmax: 983.3 RU
95% CI Kd: 4.674 to 14.04 μM
95% CI Bmax: 859.2 to 1131 RU
R squared: 0.9662
L
Replicate 2
Ligand: BMP2 (4239 RU)
Analyte: BMPR1B ECD+EDTA
Kd: 9.537 μM
Bmax: 867.6 RU
95% CI Kd: 5.727 to 15.76 μM
95% CI Bmax: 762.5 to 992.0 RU
R squared: 0.9716
Binding (RU)
Time (s)
BMPR1B ectodomain (μM)

Supplementary Fig. 5. SPR-based equilibrium binding experiments between BMPR1B ectodomain and three BMPs in the presence of calcium or EDTA.

(**A–B**) SPR-based equilibrium binding experiments between BMPR1B ectodomain and BMP7 in the presence of 2 mM $CaCl_2$. Both SPR sensorgrams and corresponding isotherms are shown. Experiments were performed in duplicate.

(**C–D**) SPR-based equilibrium binding experiments between BMPR1B ectodomain and BMP7 in the presence of 2 mM EDTA pH 8.0. Both SPR sensorgrams and corresponding isotherms are shown. Experiments were performed in duplicate.

(**E–F**) SPR-based equilibrium binding experiments between BMPR1B ectodomain and GDF5 in the presence of 2 mM $CaCl_2$. Both SPR sensorgrams and corresponding isotherms are shown. Experiments were performed in duplicate.

(**G–H**) SPR-based equilibrium binding experiments between BMPR1B ectodomain and GDF5 in the presence of 2 mM EDTA pH 8.0. Both SPR sensorgrams and corresponding isotherms are shown. Experiments were performed in duplicate.

(**I–J**) SPR-based equilibrium binding experiments between BMPR1B ectodomain and BMP2 in the presence of 2 mM $CaCl_2$. Both SPR sensorgrams and corresponding isotherms are shown. Experiments were performed in duplicate.

(**K–L**) SPR-based equilibrium binding experiments between BMPR1B ectodomain and BMP2 in the presence of 2 mM EDTA pH 8.0. Both SPR sensorgrams and corresponding isotherms are shown. Experiments were performed in duplicate.

Twelve different concentrations of the analyte (BMPR1B ectodomain (ECD)) ranging from 0.0732 µM to 150 µM were injected over SPR chip with BMP7, GDF5, or BMP2 ligands either in the presence of calcium or EDTA. The analyte concentrations associated with each sensorgram are depicted in panel **A**. Amount (RU) of immobilized ligand on the chip is indicated. K_d, equilibrium binding dissociation constant; B_{max}, maximum response at saturating concentration of analyte; 95% CI, 95% Confidence Intervals, both for K_d and B_{max}, are indicated; RU, resonance units; n/a, not applicable.

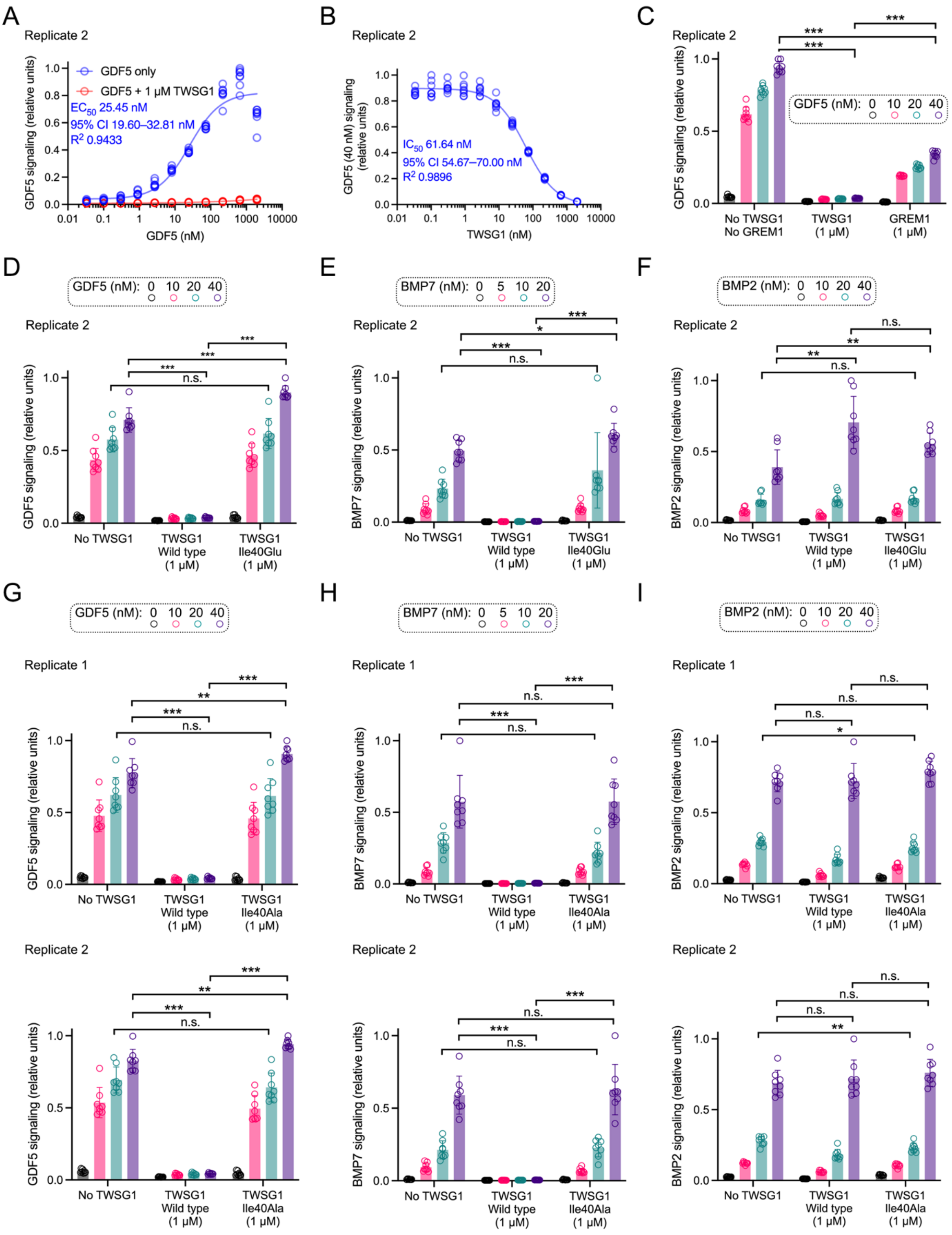
A
Replicate 2
GDF5 only
GDF5 + 1 µM TWSG1
EC50 25.45 nM
95% CI 19.60–32.81 nM
R2 0.9433
GDF5 signaling (relative units)
GDF5 (nM)
B
Replicate 2
IC50 61.64 nM
95% CI 54.67–70.00 nM
R2 0.9896
GDF5 (40 nM) signaling (relative units)
TWSG1 (nM)
C
Replicate 2
GDF5 (nM): 0 10 20 40
No TWSG1 No GREM1
TWSG1 (1 µM)
GREM1 (1 µM)
D
GDF5 (nM): 0 10 20 40
Replicate 2
GDF5 signaling (relative units)
No TWSG1
TWSG1 Wild type (1 µM)
TWSG1 Ile40Glu (1 µM)
E
BMP7 (nM): 0 5 10 20
Replicate 2
BMP7 signaling (relative units)
F
BMP2 (nM): 0 10 20 40
Replicate 2
BMP2 signaling (relative units)
G
GDF5 (nM): 0 10 20 40
Replicate 1
Replicate 2
TWSG1 Ile40Ala (1 µM)
H
BMP7 (nM): 0 5 10 20
Replicate 1
Replicate 2
I
BMP2 (nM): 0 10 20 40
Replicate 1
Replicate 2

Supplementary Fig. 6. TWSG1 inhibits GDF5 and BMP7 (but not BMP2) signaling in cellular assays.

(**A**) GDF5 activates SMAD-dependent BMP signaling in a concentration-dependent manner in C2C12 myoblasts with a half-maximal effective concentration (EC_{50}) of 25.45 nM (blue circles). TWSG1 (1 µM) inhibits GDF5 signaling (red circles). GDF5 signaling was measured eight times at each GDF5 concentration (n=8, indicated by open circles). 95% CI, 95% Confidence Interval. XY signaling data were fitted to a non-linear, sigmoidal, four parameter logistic model using GraphPad Prism.

(**B**) TWSG1 inhibits GDF5 (40 nM) signaling in a concentration-dependent manner in C2C12 myoblasts with a half-maximal inhibitory concentration (IC_{50}) of 61.64 nM. GDF5 signaling was measured eight times at each GDF5 concentration (n=8, indicated by open circles). 95% CI, 95% Confidence Interval. XY signaling data were fitted to a non-linear, sigmoidal, four parameter logistic model using GraphPad Prism.

(**C**) Comparison of TWSG1 and Gremlin 1 (GREM1), a BMP signaling inhibitor. Both TWSG1 (1 µM) and GREM1 (1 µM) inhibited GDF5 signaling (0, 10, 20, and 40 nM) but TWSG1 seemed to be a stronger inhibitor than GREM1.

(**D–E**) Wild-type TWSG1 (but not TWSG1 Ile40Glu) inhibits GDF5 (**D**) and BMP7 (**E**) signaling.

(**F**) Neither wild-type TWSG1 nor TWSG1 Ile40Glu inhibits BMP2 signaling.

(**G–H**) Wild-type TWSG1 (but not TWSG1 Ile40Ala) inhibits GDF5 (**G**) and BMP7 (**H**) signaling.

(**I**) Neither wild-type TWSG1 nor TWSG1 Ile40Ala inhibits BMP2 signaling.

Each column represents the average GDF5 signaling, measured eight times (n=8, indicated by open circles) in panels **C–I**. Experiments presented in panels **G–I** were performed in duplicate in two 96-well plates. Standard deviations are indicated by vertical T-shaped bars on each column. P values were calculated using Student's two-sample t-test, assuming unequal variance: n.s., not significant, $P > 0.05$; *, $P \leq 0.05$; **, $P \leq 0.01$; ***, $P \leq 0.001$.

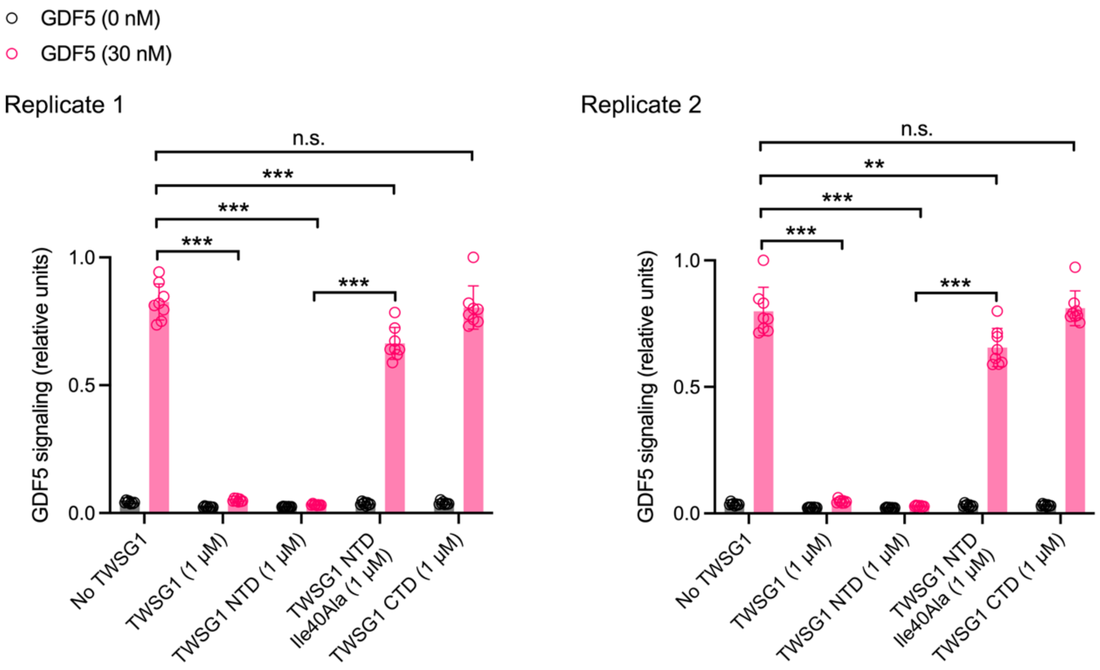

Supplementary Fig. 7. The N-terminal domain (NTD) of TWSG1 is sufficient for inhibition of GDF5 signaling in cellular assays.

GDF5 (30 nM) activates SMAD-dependent signaling in C2C12 myoblasts (first pink column). Both full-length TWSG1 (1 µM) and its N-terminal domain (NTD, 1 µM) inhibit GDF5 signaling (second and third pink columns, respectively). The mutation Ile40Ala impairs the inhibitory function of TWSG1 Ile40Ala NTD (fourth pink column), whereas TWSG1 CTD has no significant effect on GDF5 signaling (fifth pink column). Each column represents the average GDF5 signaling, measured eight times (n=8, indicated by open circles). Experiments were performed in duplicate in two 96-well plates. Standard deviations are indicated by vertical T-shaped bars on each column. P values were calculated using Student's two-sample t-test, assuming unequal variance: n.s., not significant, $P > 0.05$; *, $P \leq 0.05$; **, $P \leq 0.01$; ***, $P \leq 0.001$.

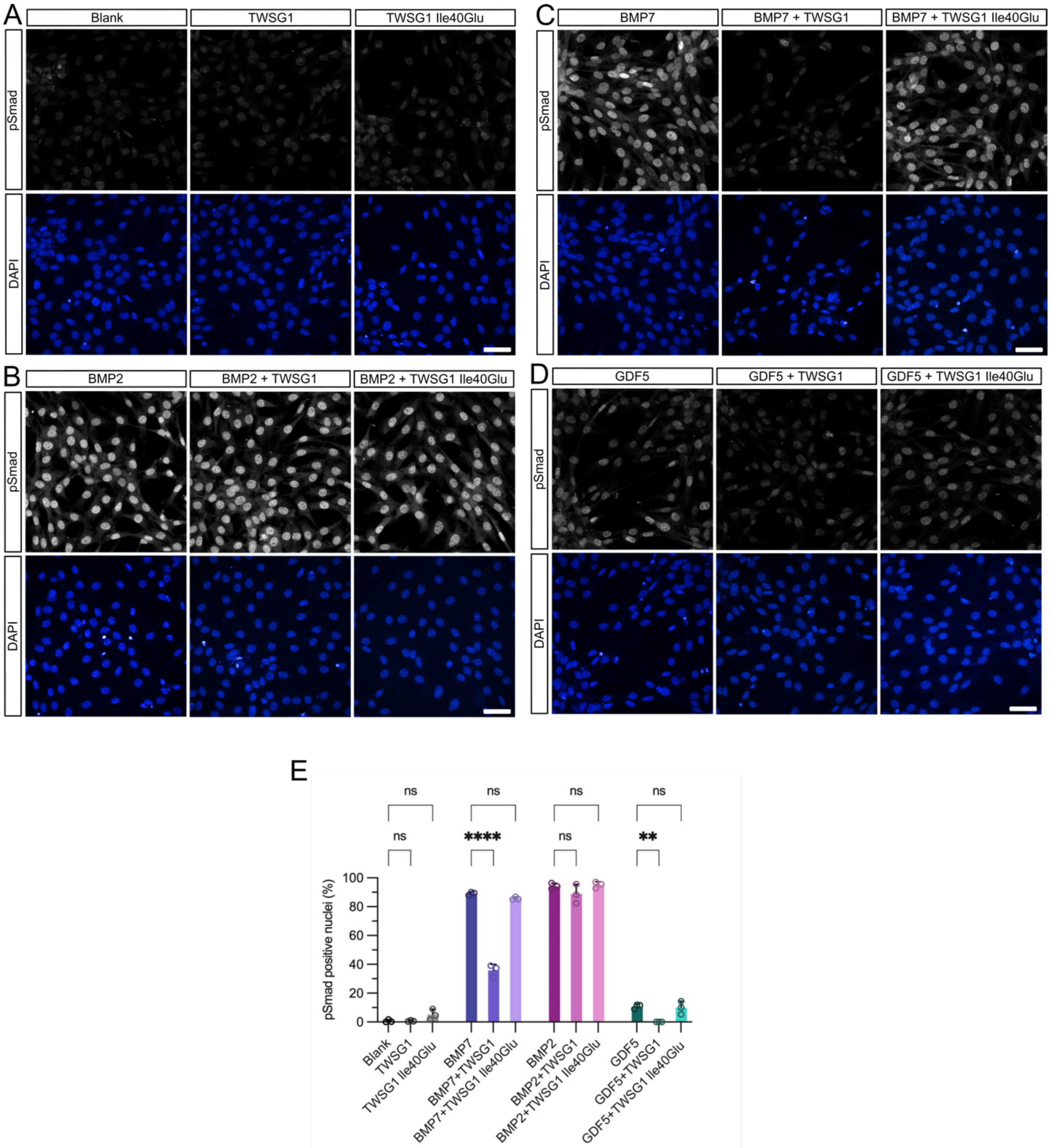

Supplementary Fig. 8. TWSG1 inhibits pSMAD signaling by BMP7 and GDF5 in C2C12 myoblast cells.

pSmad immunofluorescence of control C2C12 cells (**A**) or those stimulated with BMP2 (**B**), BMP7 (**C**) or GDF5 (**D**) ligands in the absence or presence of TWSG1 or TWSG1 Ile40Glu. DAPI nuclear staining is shown in blue. Scale bars: 50 µm. (**E**) Quantitation of pSmad positive nuclei as a percentage of total nuclei per condition. n = 3, biologically independent experiments, with 200–300 cells examined for each replicate. Error bars represent standard deviation from the mean. P values were calculated using one-way ANOVA, Šídák's multiple comparisons test: ns, $P > 0.05$; **, $P \leq 0.01$; ****, $P \leq 0.0001$ (Blank vs TWSG1 $P > 0.999$; Blank vs TWSG1 Ile40Glu $P = 0.518$; BMP7 vs BMP7+TWSG1 $P < 1.0\times10^{-15}$; BMP7 vs BMP7+TWSG1 Ile40Glu $P = 0.802$; BMP2 vs BMP2+TWSG1 $P = 0.254$; BMP2 vs BMP2+TWSG1 Ile40Glu $P = 0.999$; GDF5 vs GDF5+TWSG1 $P = 0.00175$; GDF5 vs GDF5+TWSG1 Ile40Glu $P = 0.999$).

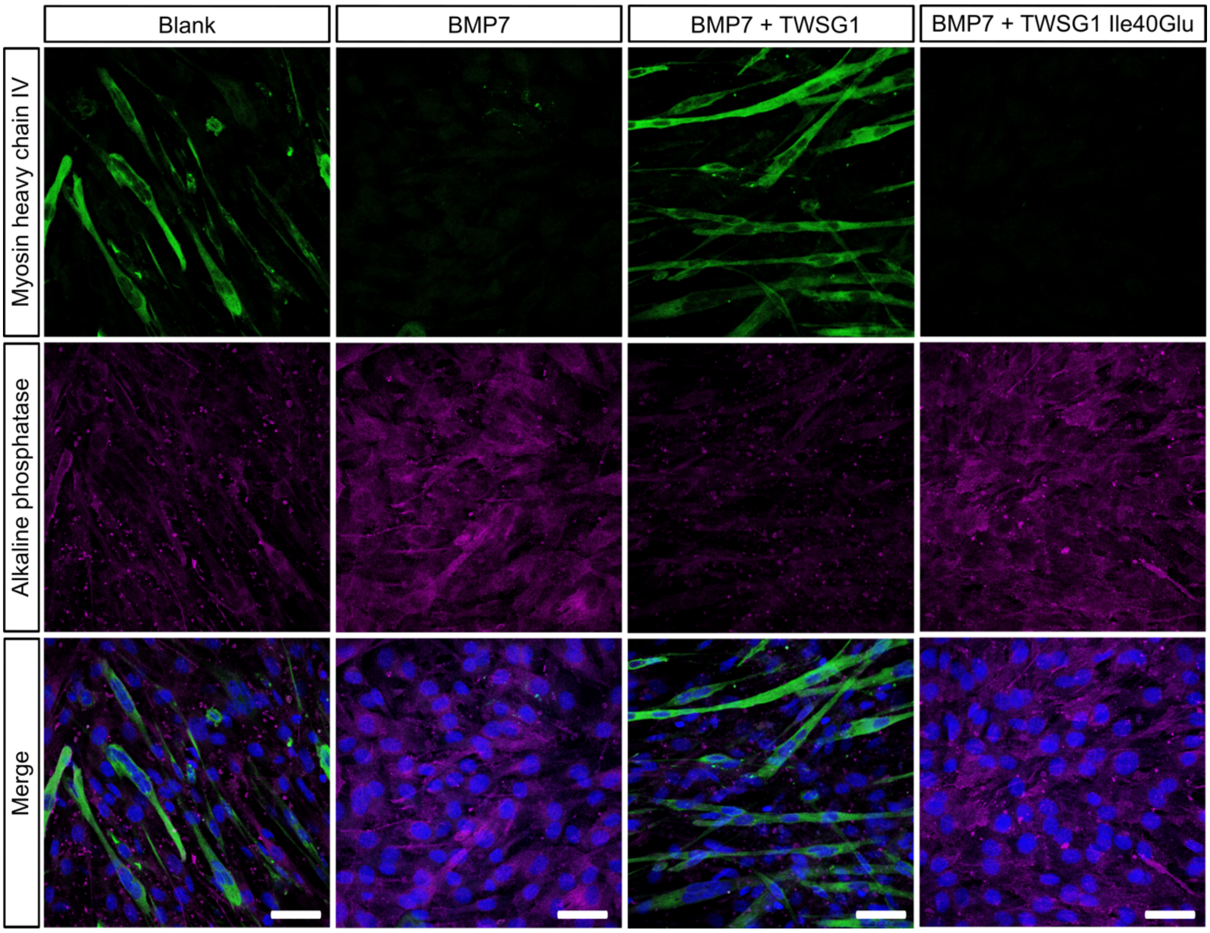

Supplementary Fig. 9. TWSG1 inhibits BMP7-directed differentiation of C2C12 myoblasts into osteoblasts in a cellular assay.

Immunostaining of control C2C12 myoblast cells (first column), or those incubated with BMP7 only (second column), and either with BMP7 plus wild type TWSG1 (third column) or with BMP7 plus TWSG1 Ile40Glu (final column) proteins. Immunostaining was performed using antibodies recognising Myosin heavy chain IV and alkaline phosphatase, which mark myotube and osteoblast fates, respectively. Wild type TWSG1, but not TWSG1 Ile40Glu, inhibits the ability of BMP7 to promote differentiation of C2C12 myoblasts into osteoblasts. n=3 biologically independent experiments. Scale bars: 50 µm.

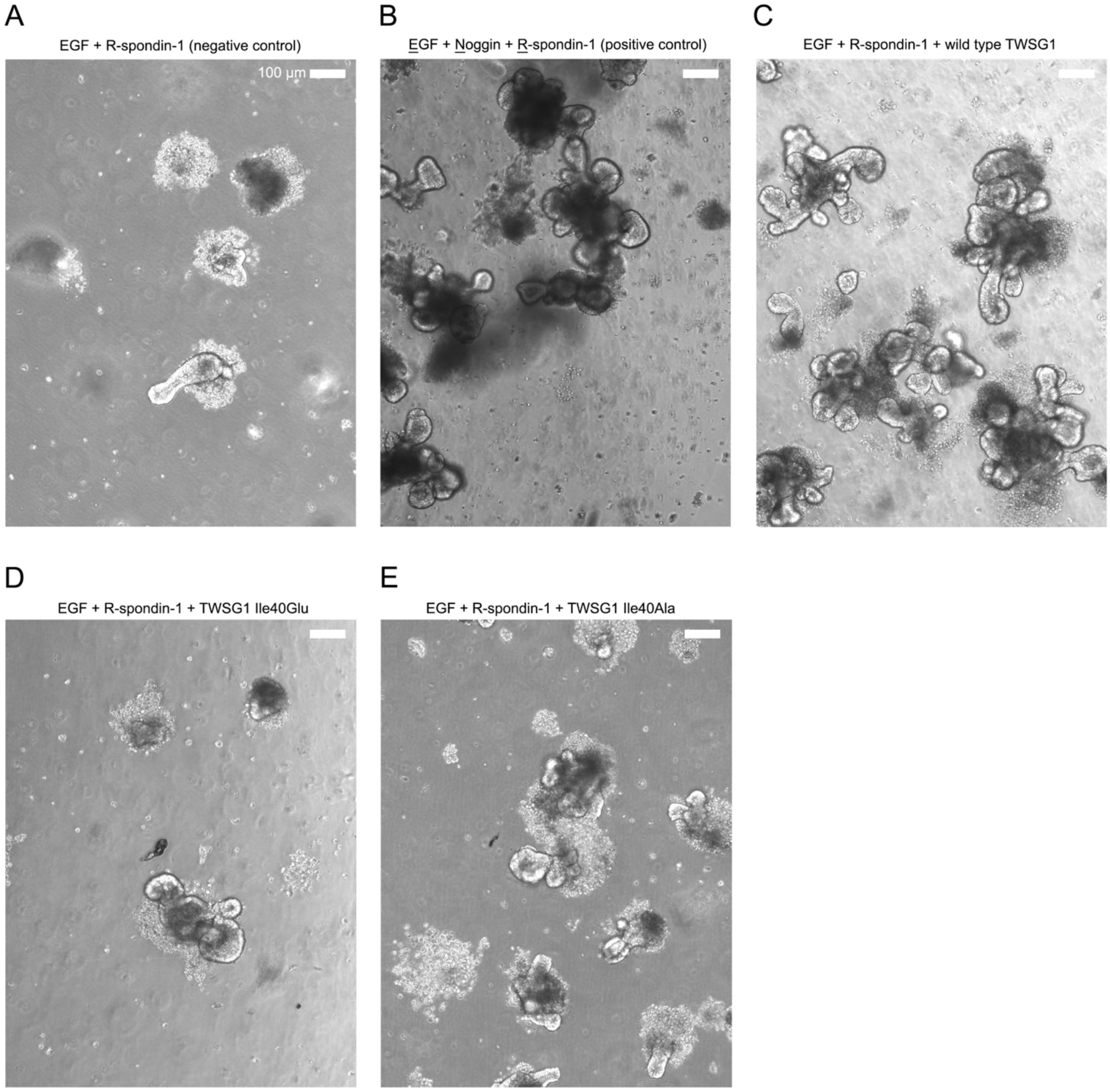

Supplementary Fig. 10. TWSG1 inhibits BMP/GDF signaling in mouse intestinal organoids. (**A–E**) Uncropped images of organoids presented in **Fig. 3I–L**. In addition, organoids grown in the presence of EGF, R-spondin-1, and TWGS1 Ile40Ala are shown in panel **E**. Scale bars: 100 µm.

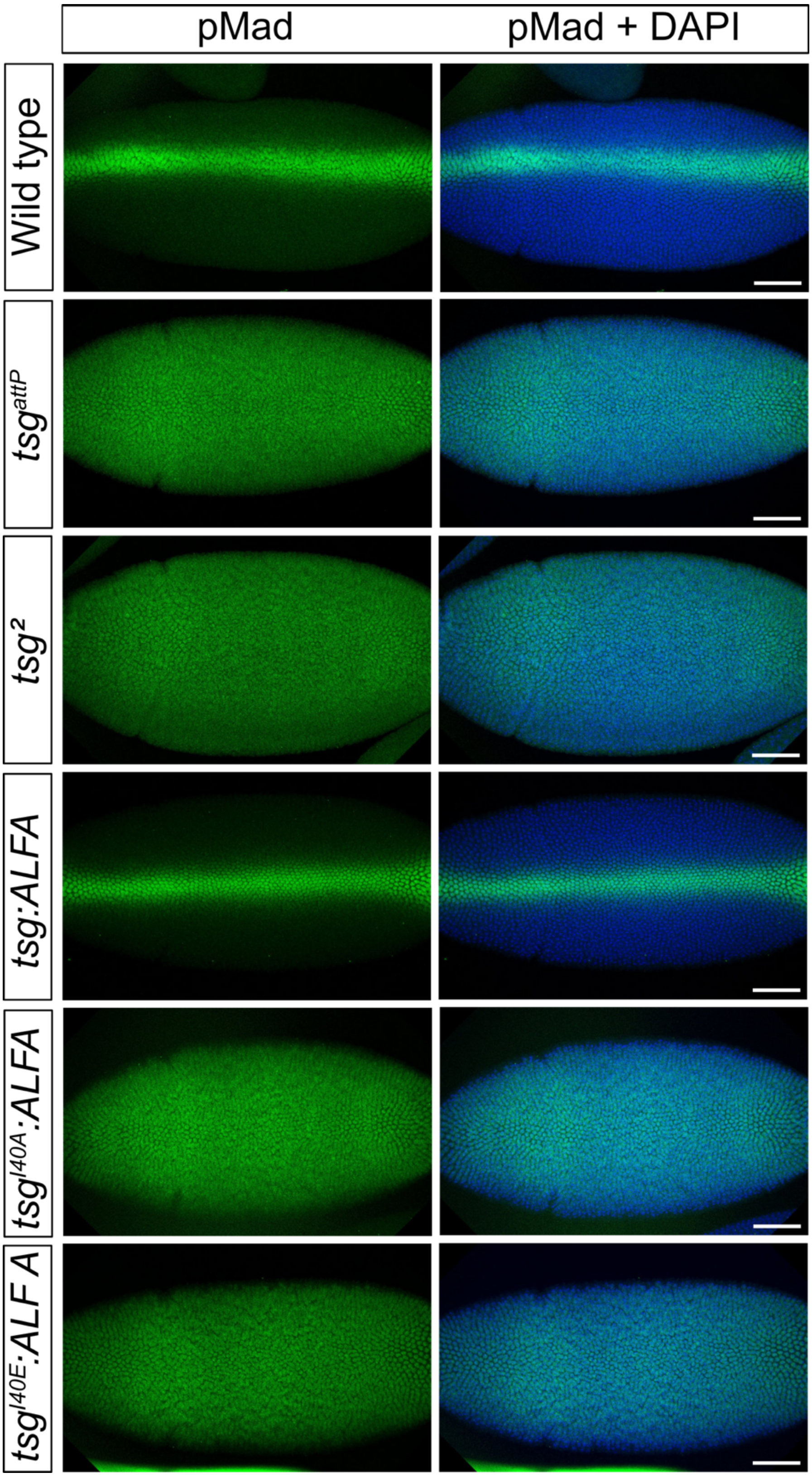

Supplementary Fig. 11. Tsg mutations of Ile40 to Ala or Glu lead to a loss of BMP gradient formation in *Drosophila* embryos as visualized by pMad staining. pMad staining in wild type, *tsg*^{2}, *tsg*^{attP}, *tsg:ALFA* and *tsgI40:ALFA* mutant embryos at the onset of gastrulation. Nuclei are stained with DAPI (blue). Scale bars: 50 µm. n=5 biologically independent animals/embryos for all genotypes with representative images presented.

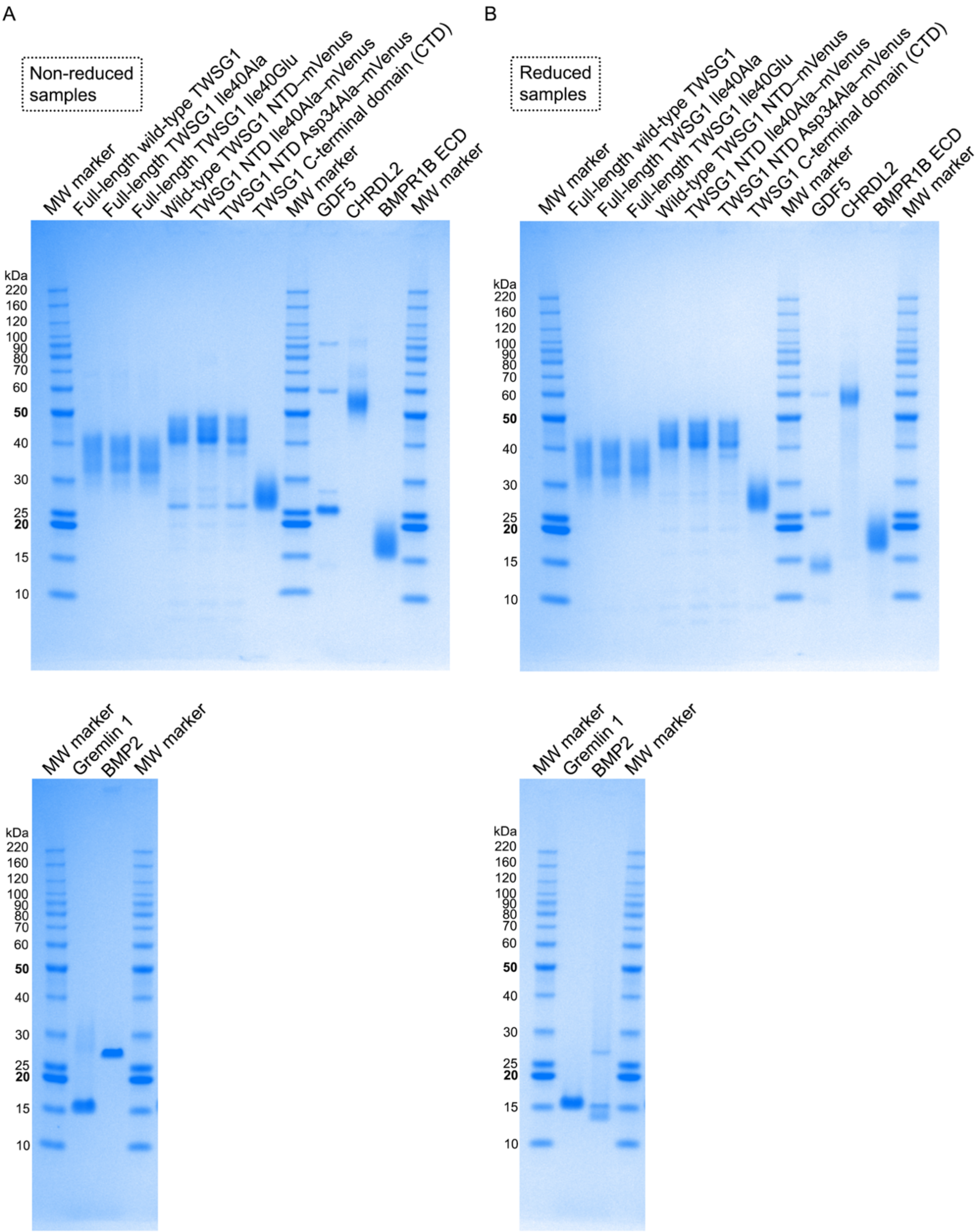

Supplementary Fig. 12. SDS-PAGE analysis of proteins used in this study. The samples were heated for 5 minutes at 100 °C before loading onto the gel. The protein samples shown in panel **A** did not contain a reducing reagent. The proteins shown in panel **B** were reduced with 2% v/v 2-mercaptoethanol. Each well contained 1 µg of purified protein. MW marker, 5 µl/well of BenchMark Protein Ladder from Thermo Fisher Scientific. SDS-PAGE was performed using NuPAGE Bis-Tris 4–12% gel in SDS-MES running buffer (Invitrogen) and stained with InstantBlue Coomassie protein stain (Expedeon). All proteins, except GDF5, BMP2 and Gremlin 1, were produced as secreted proteins via transient expression of HEK293T cells and include full-length glycans.

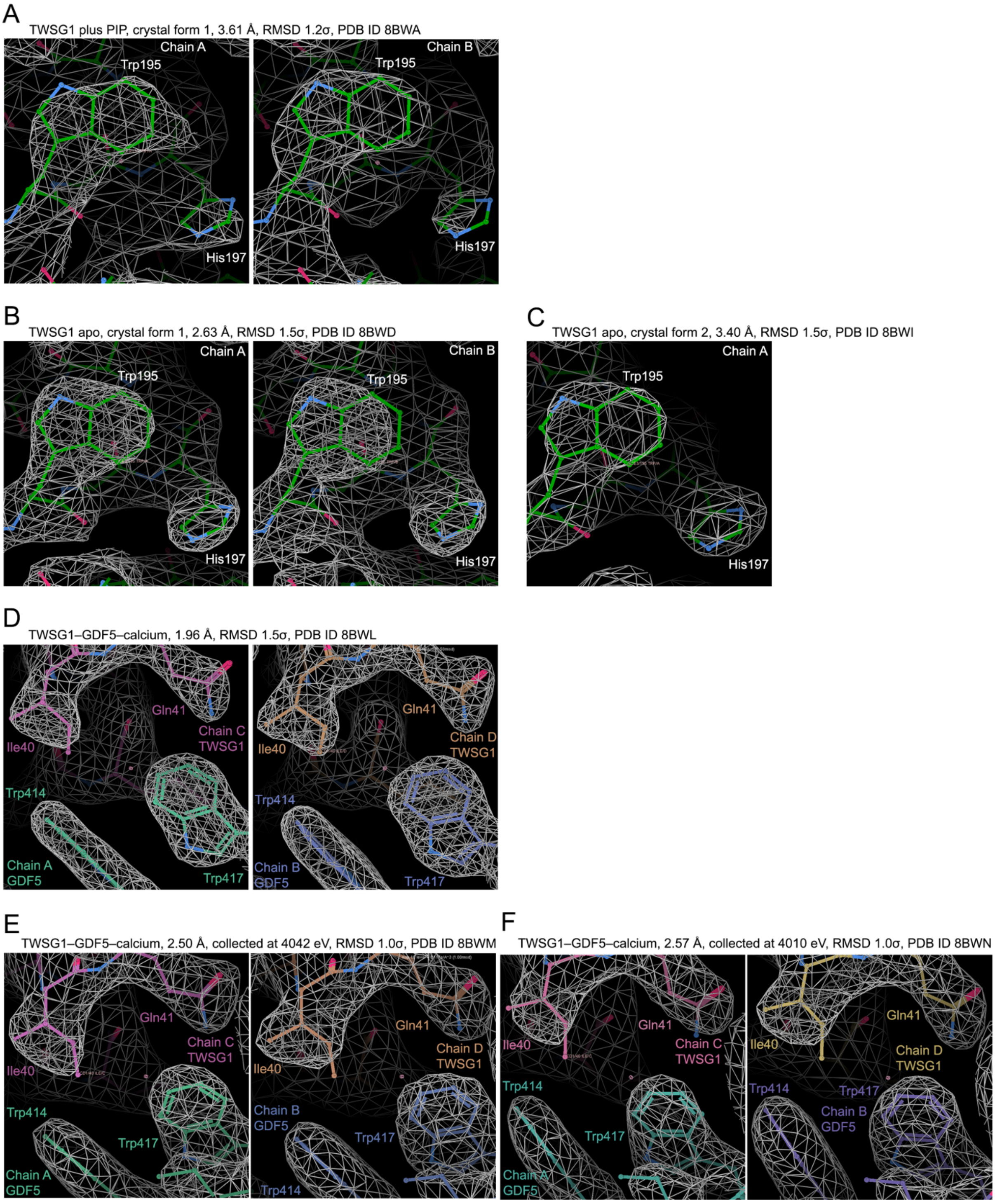

Supplementary Fig. 13. Snapshots of electron density maps of the six crystal structures presented in this study. 2F_o-F_c electron density maps were calculated using phenix.refine and visualized as white mesh at the 1.0σ–1.5σ contour level (indicated in each panel) in Coot. In panel **A**, B-factors were sharpened (-200) in Coot. In panels **A** and **B**, snapshots of two TWSG1 molecules in the asymmetric unit are shown. In panel **C**, a snapshot of a single TWSG1 molecule present in the asymmetric unit is shown. In panels **D**–**F**, snapshots of two TWSG1 NTD–GDF5 interfaces are displayed. The resolution of each crystal structure and Protein Data Bank (PDB) ID codes are indicated in each panel and correspond to **Supplementary Table 1**.

Supplementary Table 1. Crystallization, X-ray data collection, processing and model refinement statistics.

	**TWSG1+PIP Crystal form 1**	**TWSG1 Crystal form 1**	**TWSG1 Crystal form 2**	**GDF5+TWSG1+ Calcium, native**	**GDF5+TWSG1+ Calcium**	**GDF5+TWSG1+ Calcium**
**PDB ID**	8BWA	8BWD	8BWI	8BWL	8BWM	8BWN
**Crystallization**						
Reservoir conditions	0.77 M LiCl, 15.5% w/v PEG 6000, 77 mM HEPES pH 7.0, 5.2% 2,2,2-trifluoroethanol	0.85 M LiCl, 17.1% w/v PEG 6000, 85 mM HEPES pH 7.0, 3.6% 1,1,1,3,3,3-hexafluoro-2-propanol	1.0 M K/Na tartrate, 0.1 M MES pH 6.0	12.1% w/v PEG 1000, 12.1% w/v PEG 3350, 12.1% v/v MPD, 97 mM $CaCl_2$, 0.097 M Bicine/Trizma pH 8.5	12.5% w/v PEG 1000, 12.5% w/v PEG 3350, 12.5% v/v MPD, 100 mM $CaCl_2$, 0.1 M MOPS/HEPES-Na pH 7.5	12.5% w/v PEG 1000, 12.5% w/v PEG 3350, 12.5% v/v MPD, 100 mM $CaCl_2$, 0.1 M MOPS/HEPES-Na pH 7.5
Protein conc. (mg/ml)	6.2	6.2	5.6	20	20	20
Protein+reservoir volume (nl)	200+100	100+100	100+100	200+100	200+100	200+100
Cryoprotectant	25% glycerol	25% glycerol	25% ethylene glycol	25% ethylene glycol	25% ethylene glycol	25% ethylene glycol
**Data collection**						
X-ray source	Diamond Light Source, I03	Diamond Light Source, I03	Diamond Light Source, I24	Diamond Light Source, I03	Diamond Light Source, I23	Diamond Light Source, I23
Space group	$P\ 6_5\ 2\ 2$	$P\ 6_5\ 2\ 2$	$P\ 6_4\ 2\ 2$	$P\ 2_1\ 2_1\ 2_1$	$P\ 2_1\ 2_1\ 2_1$	$P\ 2_1\ 2_1\ 2_1$
Unit cell dimensions *a*, *b*, *c* (Å)	98.430, 98.430, 188.160	97.993, 97.993, 187.616	92.10, 92.10, 96.16	57.047, 88.588, 97.622	57.480, 89.263, 97.782	57.574, 89.178, 97.681
Unit cell angles α, β, γ (°)	90, 90, 120	90, 90, 120	90, 90, 120	90, 90, 90	90, 90, 90	90, 90, 90
Wavelength (Å)	1.0300	0.97625	0.96862	0.97625	3.06740 (4042 eV)	3.09187 (4010 eV)
Resolution (Å)	62.72–3.61 (3.67–3.61)	84.86–2.63 (3.00–2.63)	48.08–3.40 (3.49–3.40)	49.25–1.96 (2.10–1.96)	65.93–2.50 (2.81–2.50)	65.86–2.57 (2.85–2.57)
R_{merge} (I) (%)	14.3 (761.2)	7.8 (160.3)	25.1 (580.6)	6.4 (193.6)	11.9 (150.7)	9.2 (159.0)
R_{meas} (I) (%)	14.6 (776.7)	8.0 (164.8)	26.7 (618.4)	6.7 (201.1)	12.6 (158.9)	9.7 (167.4)
R_{pim} (I) (%)	2.9 (152.4)	1.9 (37.7)	6.9 (155.1)	1.9 (54.2)	3.8 (49.9)	2.9 (52.0)
Completeness (%)	99.5 (99.7)	94.0 (ellipsoidal) (74.8), 53.4 (spherical) (8.4)	99.4 (98.5)	94.7 (ellipsoidal) (53.1), 84.2 (spherical) (22.5)	92.8 (ellipsoidal) (57.6), 68.3 (spherical) (11.7)	92.9 (ellipsoidal) (57.0), 72.0 (spherical) (13.9)
I/σ(*I*)	13.6 (0.6)	22.0 (1.9)	8.2 (0.6)	17.9 (1.4)	12.2 (1.4)	13.5 (1.3)
Completeness (%)	99.5 (99.7)	94.0 (ellipsoidal) (74.8), 53.4 (spherical) (8.4)	99.4 (98.5)	94.7 (ellipsoidal) (53.1), 84.2 (spherical) (22.5)	92.8 (ellipsoidal) (57.6), 68.3 (spherical) (11.7)	92.9 (ellipsoidal) (57.0), 72.0 (spherical) (13.9)
Multiplicity	25.8 (25.4)	18.8 (18.7)	15.6 (15.4)	13.1 (13.7)	10.0 (9.9)	10.2 (10.1)
Anomalous completeness (%)	98.9 (99.3)				92.8 (57.6) (ellipsoidal) 68.3 (11.7) (spherical)	92.1 (56.1) (ellipsoidal) 70.7 (13.6) (spherical)
Anomalous multiplicity (%)	14.8 (13.8)				92.8 (57.6) (ellipsoidal) 68.3 (11.7) (spherical)	92.1 (56.1) (ellipsoidal) 70.7 (13.6) (spherical)
Anomalous correlation (%)	90.5 (9.7)				58.0 (-0.02)	51.6 (1.2)
**Refinement**						
Resolution (Å)	49.22–3.61	42.43–2.63	46.12–3.40	49.25–1.96	65.92–2.50	65.86–2.57
No. reflections	6554	8846	2934	30651	12359	12003
R_{work}/R_{free} (%)	33.9/39.0	25.9/30.5	30.0/34.1	18.4/21.2	22.6/27.1	25.2/28.3
No. of atoms	4817	4816	2444	4821	4720	4708
Protein	4810	4810	2444	4664	4646	4660
Ligand/ion	7 (Pt)	5 (SO_4^{2-})	0	4 (Ca)	3 (Ca)	3 (Ca)
Water	0	1	0	153	71	45
B factors (Å^2), All	246	101	116	70	90	94
Protein	246	101	116	71	91	95
Ligand/ion	7	133	N/A	98	106	111
Water	N/A	68	N/A	63	70	75
RMSD bond lengths (Å)/angles (°)	0.002/0.596	0.003/0.652	0.003/0.695	0.017/1.365	0.002/0.479	0.002/0.411
Ramachandran (%) outliers	0.00	0.00	0.0	0.0	0.00	0.00
favored	94.4	93.4	92.6	95.0	96.6	96.0
MolProbity overall score	1.49	1.62	1.44	1.31	1.03	1.09
MolProbity clash score	3.11	3.95	2.05	1.93	1.08	1.07

Values in parentheses are for the highest-resolution shell. RMSD, root-mean-square deviations from ideal values. N/A, not applicable.

Supplementary Table 2. Cell lines and plasmids used in this study.

Cells and plasmids	Reference/Source	Catalog number or other identifier
Drosophila, mouse and human cell lines		
C2C12 immortalized mouse myoblasts stably transfected with a reporter plasmid of BMP signaling	[5]	Not applicable
Human embryonic kidney (HEK) 293T cells	ATCC	Cat # CRL-11268
Drosophila S2R+	[6]	Not applicable
Drosophila Stocks		
D. melanogaster; y^1w^{67c23}	Bloomington *Drosophila* Stock Centre	L6599
D. melanogaster; y[1] sc[*] v[1,] sev[21]; P{y[+t7.7] v[+t1.8]=nos-Cas9.R}attP2	Bloomington *Drosophila* Stock Centre	BL78782
D. melanogaster; y1 w67c23; snaSco/CyO, P{w+mC=Crew}DH1	Bloomington *Drosophila* Stock Centre	BL1092
D. melanogaster; w[*] tsg[2]/FM7c	Bloomington *Drosophila* Stock Centre	BL2187
*D. melanogaster; w*tsg^2/FM7c-ftz-lacZ*	This study	Not applicable
D. melanogaster; brkM68/FM7c-ftz-lacZ	[7]	Not applicable
Plasmids		
pHD-DsRed-attP	[8]	Addgene # 51019
pHD-DsRed-attP-HA1HA2	This manuscript	Not applicable
pET22b-GDF5	This manuscript	UniProt ID P43026 (Ala382–Arg501)
pET22b-Gremlin-1	This manuscript	UniProt ID O60565 (Val73–Asp184)
pET28-His$_6$-tagged HRV 3C protease	[9]	UniProt ID P03303, Gly1538–Gln1719
pGEX-3X-GST-tagged endoglycosidase F1	[10]	NCBI ref. seq WP_034866176.1 (Ala51–Trp339)
pHLsec-CHRDL2	This manuscript	UniProt ID Q6WN34 (Ala25–Thr429)
pHLsec-full-length TWSG1	This manuscript	UniProt ID Q9GZX9 (Cys26–Phe223)
pHLsec-full-length TWSG1 Ile40Ala	This manuscript	UniProt ID Q9GZX9 (Cys26–Phe223, Ile40Ala)
pHLsec-full-length TWSG1 Ile40Glu	This manuscript	UniProt ID Q9GZX9 (Cys26–Phe223, Ile40Glu)
pHR-CMV-TetO2-TWSG1$_{CTD}$-His$_6$	This manuscript	UniProt ID Q9GZX9 (Thr85–Phe223)
pHR-CMV-TetO2-TWSG1$_{NTD}$-3C-mVenus-His$_{12}$	This manuscript	UniProt ID Q9GZX9 (Cys26–Ser83)
pHR-CMV-TetO2-TWSG1$_{NTD}$-3C-mVenus-His$_{12}$ Ile40Ala	This manuscript	UniProt ID Q9GZX9 (Cys26–Ser83, Ile40Ala)
pHR-CMV-TetO2-TWSG1$_{NTD}$-3C-mVenus-His$_{12}$ Ile40Glu	This manuscript	UniProt ID Q9GZX9 (Cys26–Ser83, Ile40Glu)
pU6-Bbs1-gRNA	[8]	Addgene # 45946

pU6-Bbs1-5’gRNA	This manuscript	Not applicable
pU6-Bbs1-3’gRNA	This manuscript	Not applicable
RIV^{white}	J.P. Vincent lab at the Francis Crick Institute[11]	DGRC_ 1330
RIV^{white}-template	This manuscript	Not applicable
RIV^{white}-Tsg:ALFA	This manuscript	Not applicable
RIV^{white}-Tsg^{I40A}:ALFA	This manuscript	Not applicable
RIV^{white}-Tsg^{I40E}:ALFA	This manuscript	Not applicable
pMT-Dpp:HA	[6]	Not applicable
pMT-Scw:Flag	[6]	Not applicable
pMT-BiP-Sog:Myc	[6]	Not applicable
pMT-BiP-Tsg:His	[6]	Not applicable
pMT-BiP-Tsg^{I40E}:His	This manuscript	Not applicable
Antibodies		
Rabbit anti-Smad3 ((phospho S423+425)[EP823Y] PUR)	Abcam	Research Resource Identifier (RRID) AB_882596
Donkey anti-rabbit IgG Alexa Fluor 647	Invitrogen	RRID AB_2536183
Rabbit anti-ALPL	Thermo Fisher Scientific	RRID AB_2722857
Mouse anti-MHC IV Alexa Fluor 488 conjugated	Thermo Fisher Scientific	RRID AB_10671272
Sheep anti-digoxigenin-AP, Fab fragments, AP conjugated	Roche	Cat # 11333089001, RRID AB_514496
Donkey anti-Sheep IgG Alexa Fluor 488	Thermo Fisher Scientific	Cat # A-11015
Rabbit anti-His	Cell Signalling Technology	Cat # 12698S
Mouse anti-Myc	Millipore	RRID AB_11211891
Chicken anti-HA	Abcam	Cat # ab9111, RRID AB_307020
Donkey anti-Rabbit IgG IRDye 680RD	LI-COR	RRID AB_2716687
Donkey anti-Mouse IgG IRDye 800CW	LI-COR	RRID AB_621847
Donkey anti-Chicken IgG IRDye 800CW	LI-COR	RRID AB_1850023

SUPPLEMENTARY REFERENCES

1. Chen J, Sawyer N, Regan L. Protein-protein interactions: general trends in the relationship between binding affinity and interfacial buried surface area. *Protein Sci* **22**, 510–515 (2013).

2. Jumper J*, et al.* Highly accurate protein structure prediction with AlphaFold. *Nature* **596**, 583–589 (2021).

3. Shimmi O, Ralston A, Blair SS, O'Connor MB. The *crossveinless* gene encodes a new member of the Twisted gastrulation family of BMP-binding proteins which, with Short gastrulation, promotes BMP signaling in the crossveins of the *Drosophila* wing. *Dev Biol* **282**, 70–83 (2005).

4. Bonds M, Sands J, Poulson W, Harvey C, Von Ohlen T. Genetic screen for regulators of *ind* expression identifies *shrew* as encoding a novel twisted gastrulation-like protein involved in Dpp signaling. *Dev Dyn* **236**, 3524–3531 (2007).

5. Herrera B, Inman GJ. A rapid and sensitive bioassay for the simultaneous measurement of multiple bone morphogenetic proteins. Identification and quantification of BMP4, BMP6 and BMP9 in bovine and human serum. *BMC Cell Biol* **10**, 20 (2009).

6. Sawala A, Sutcliffe C, Ashe HL. Multistep molecular mechanism for bone morphogenetic protein extracellular transport in the *Drosophila* embryo. *Proc Natl Acad Sci U S A* **109**, 11222–11227 (2012).

7. Jaźwińska A, Rushlow C, Roth S. The role of brinker in mediating the graded response to Dpp in early *Drosophila* embryos. *Development* **126**, 3323–3334 (1999).

8. Gratz SJ*, et al.* Highly specific and efficient CRISPR/Cas9-catalyzed homology-directed repair in *Drosophila*. *Genetics* **196**, 961–971 (2014).

9. Robinson RA*, et al.* Simultaneous binding of Guidance Cues NET1 and RGM blocks extracellular NEO1 signaling. *Cell* **184**, 2103–2120 e31 (2021).

10. Grueninger-Leitch F, D'Arcy A, D'Arcy B, Chène C. Deglycosylation of proteins for crystallization using recombinant fusion protein glycosidases. *Protein Science* **5**, 2617–2622 (1996).

11. Baena-Lopez LA, Alexandre C, Mitchell A, Pasakarnis L, Vincent JP. Accelerated homologous recombination and subsequent genome modification in *Drosophila*. *Development* **140**, 4818–4825 (2013).
