# Supplementary material for: Molecular mechanism of BMP signal control by Twisted gastrulation
Source: Nat Commun. 2024 Jun 11;15:4976. doi: 10.1038/s41467-024-49065-8 (PMC11167000; doi:10.1038/s41467-024-49065-8)
Supplement: Supplementary file 3 — Reporting Summary [file 41467_2024_49065_MOESM3_ESM.pdf]

nature portfolio

Corresponding author(s): Tomas Malinauskas
Hilary L. Ashe
Christian Siebold

Last updated by author(s): Apr 15, 2024

Reporting Summary

Nature Portfolio wishes to improve the reproducibility of the work that we publish. This form provides structure for consistency and transparency in reporting. For further information on Nature Portfolio policies, see our Editorial Policies and the Editorial Policy Checklist.

Statistics

For all statistical analyses, confirm that the following items are present in the figure legend, table legend, main text, or Methods section.

n/a	Confirmed	
☐	☒	The exact sample size (n) for each experimental group/condition, given as a discrete number and unit of measurement
☐	☒	A statement on whether measurements were taken from distinct samples or whether the same sample was measured repeatedly
☐	☒	The statistical test(s) used AND whether they are one- or two-sided<br>*Only common tests should be described solely by name; describe more complex techniques in the Methods section.*
☒	☐	A description of all covariates tested
☒	☐	A description of any assumptions or corrections, such as tests of normality and adjustment for multiple comparisons
☐	☒	A full description of the statistical parameters including central tendency (e.g. means) or other basic estimates (e.g. regression coefficient) AND variation (e.g. standard deviation) or associated estimates of uncertainty (e.g. confidence intervals)
☐	☒	For null hypothesis testing, the test statistic (e.g. F, t, r) with confidence intervals, effect sizes, degrees of freedom and P value noted<br>*Give P values as exact values whenever suitable.*
☒	☐	For Bayesian analysis, information on the choice of priors and Markov chain Monte Carlo settings
☒	☐	For hierarchical and complex designs, identification of the appropriate level for tests and full reporting of outcomes
☒	☐	Estimates of effect sizes (e.g. Cohen's d, Pearson's r), indicating how they were calculated

Our web collection on statistics for biologists contains articles on many of the points above.

Software and code

Policy information about availability of computer code

Data collection	BIAevaluation 3.0 (Biacore/Cytiva) - SPR<br>UNICORN (Cytiva) - Size-exclusion chromatography (SEC)<br>ASTRA 6 (Wyatt Technologies) - Size-exclusion chromatography coupled to multi-angle light scattering (SEC-MALS)<br>EVOS M5000 imaging system (Invitrogen) - imaging of organoids<br>Huygens Remote Manager 3.7.1 - imaging of fly embryos<br>Fiji Image J - processing of images with fly embryos
Data analysis	Prism 9.4.1 from GraphPad - graphs representing SPR, SEC-MALS and cell signaling data<br>xia2 (https://xia2.github.io/index.html), XDS (https://xds.mr.mpg.de/html_doc/downloading.html), AIMLESS (https://www.ccp4.ac.uk/download/), STARANISO (https://staraniso.globalphasing.org/cgi-bin/staraniso.cgi), autoSHARP (https://www.globalphasing.com/sharp/), Phaser (https://www.ccp4.ac.uk/download/), CHOOCH (https://sourceforge.net/projects/chooch/), Coot (http://www2.mrc-lmb.cam.ac.uk/personal/pemsley/coot/), Phenix (https://www.phenix-online.org/download/) - X-ray data processing and structure determination<br>MolProbity (http://molprobity.biochem.duke.edu/) - structure validation<br>PDBsum (http://www.ebi.ac.uk/pdbsum) - analysis of TWSG1-GDF5 interaction interfaces<br>PyMOL from Schrödinger, LLC (https://www.pymol.org/) - visualization of protein structures<br>LAS X from Leica Microsystems Inc(https://www.cellularimaging.nl/leica-las-x/) - analysis of images with fly embryos<br>Peer-reviewed references are provided in the manuscript where applicable.

For manuscripts utilizing custom algorithms or software that are central to the research but not yet described in published literature, software must be made available to editors and reviewers. We strongly encourage code deposition in a community repository (e.g. GitHub). See the Nature Portfolio guidelines for submitting code & software for further information.

Data

Policy information about availability of data

All manuscripts must include a data availability statement. This statement should provide the following information, where applicable:

- Accession codes, unique identifiers, or web links for publicly available datasets
- A description of any restrictions on data availability
- For clinical datasets or third party data, please ensure that the statement adheres to our policy

Coordinates and structure factors have been deposited in the Protein Data Bank with the following accession numbers: 8BWA [http://doi.org/10.2210/pdb8BWA/pdb] (TWSG1+PIP, crystal form 1), 8BWD [http://doi.org/10.2210/pdb8BWD/pdb] (TWSG1, crystal form 1), 8BWI [http://doi.org/10.2210/pdb8BWI/pdb] (TWSG1, crystal form 2), 8BWL [http://doi.org/10.2210/pdb8BWL/pdb] (GDF5+TWSG1+calcium, native), 8BWM [http://doi.org/10.2210/pdb8BWM/pdb] (GDF5+TWSG1 +calcium, 4042 eV), and 8BWN [http://doi.org/10.2210/pdb8BWN/pdb] (GDF5+TWSG1+calcium, 4010 eV). All other data needed to evaluate the conclusions in the paper are present in the paper, the Supplementary Information and Source Data file.

Research involving human participants, their data, or biological material

Policy information about studies with human participants or human data. See also policy information about sex, gender (identity/presentation), and sexual orientation and race, ethnicity and racism.

Reporting on sex and gender	N/A
Reporting on race, ethnicity, or other socially relevant groupings	N/A
Population characteristics	N/A
Recruitment	N/A
Ethics oversight	N/A

Note that full information on the approval of the study protocol must also be provided in the manuscript.

Field-specific reporting

Please select the one below that is the best fit for your research. If you are not sure, read the appropriate sections before making your selection.

[x] Life sciences [ ] Behavioural & social sciences [ ] Ecological, evolutionary & environmental sciences

For a reference copy of the document with all sections, see nature.com/documents/nr-reporting-summary-flat.pdf

Life sciences study design

All studies must disclose on these points even when the disclosure is negative.

Sample size	Sample sizes of cells, organoids, and Drosophila embryos are indicated in the manuscript. No statistical methods were used to predetermine sample size.
Data exclusions	No data were excluded from the analyses.
Replication	Two replicate experiments of cellular signaling assays in C2C12 cells and two replicate SPR-based binding assays were performed. Data from replicate experiments are presented in the Supplementary Information file.
Randomization	We did not use randomization for sample allocation in our experiments, which included protein production, crystallization, X-ray data collection, determination of crystal structures and analysis, cellular signaling assays, SPR-based binding assays, as well as structure-guided experiments in Drosophila embryos. This approach is primarily because these specific types of experiments do not necessitate randomization.
Blinding	The authors were not blinded. In the context of our research, blinding during data collection and analysis was neither feasible nor relevant. Specifically, for X-ray crystallographic analysis, the objective is to determine the structure of the sample, and providing sample details beforehand is essential.

Reporting for specific materials, systems and methods

We require information from authors about some types of materials, experimental systems and methods used in many studies. Here, indicate whether each material, system or method listed is relevant to your study. If you are not sure if a list item applies to your research, read the appropriate section before selecting a response.

Materials & experimental systems

n/a	Involved in the study	
	x	Antibodies
	x	Eukaryotic cell lines
x		Palaeontology and archaeology
	x	Animals and other organisms
x		Clinical data
x		Dual use research of concern
x		Plants

Methods

n/a	Involved in the study	
x		ChIP-seq
x		Flow cytometry
x		MRI-based neuroimaging

Antibodies

Antibodies used

Antibodies are listed in Supplementary Table 2 and below:
1.
Rabbit anti-Smad3 ((phospho S423+425)[EP823Y] PUR)
Abcam
https://www.abcam.com/products/primary-antibodies/smad3-phospho-s423--s425-antibody-ep823y-ab52903.html
Research Resource Identifier (RRID) AB_882596
2.
Donkey anti-rabbit IgG Alexa Fluor 647
Invitrogen/Thermo Fisher Scientific
https://www.thermofisher.com/antibody/product/Donkey-anti-Rabbit-IgG-H-L-Highly-Cross-Adsorbed-Secondary-Antibody-Polyclonal/A-31573
RRID AB_2536183
3.
ALPL Recombinant Rabbit Monoclonal Antibody (7H11L3)
Invitrogen/Thermo Fisher Scientific
https://www.thermofisher.com/antibody/product/ALPL-Antibody-clone-7H11L3-Recombinant-Monoclonal/702454
RRID AB_2722857
4.
Mouse anti-MHC IV Alexa Fluor 488
Invitrogen/Thermo Fisher Scientific
https://tfcom-global-nginx.commerceprod.thermofisher.com/antibody/product/Myosin-4-Antibody-clone-MF20-Monoclonal/53-6503-82
RRID AB_10671272
5.
Sheep anti-digoxigenin-AP, Fab fragments, AP conjugated
Roche
https://www.sigmaaldrich.com/GB/en/product/roche/11333089001
RRID AB_514496
6.
Donkey anti-Sheep IgG Alexa Fluor 488
https://www.thermofisher.com/antibody/product/Donkey-anti-Sheep-IgG-H-L-Cross-Adsorbed-Secondary-Antibody-Polyclonal/A-11015
Thermo Fisher Scientific Catalogue # A-11015
7.
Rabbit anti-His
Cell Signaling Technolog
https://www.cellsignal.com/products/primary-antibodies/his-tag-d3i1o-xp-rabbit-mab/12698
Catalogue number 12698S
8.
Mouse anti-Myc
Millipore
https://www.merckmillipore.com/GB/en/product/Anti-Myc-Tag-Antibody-clone-4A6,MM_NF-05-724
RRID AB_11211891
9.
Chicken anti-HA
Abcam
https://www.abcam.com/products/primary-antibodies/ha-tag-antibody-ab9111.html
RRID AB_307020
10.
Donkey anti-Rabbit IgG IRDye 680RD
LI-COR
https://www.licor.com/bio/reagents/irdye-680rd-donkey-anti-rabbit-igg-secondary-antibody
RRID AB_2716687

11.
Donkey anti-Mouse IgG IRDye 800CW
LI-COR
https://www.licor.com/bio/reagents/irdye-800cw-donkey-anti-mouse-igg-secondary-antibody
RRID AB_621847
12.
Donkey anti-Chicken IgG IRDye 800CW
https://www.licor.com/bio/reagents/irdye-800cw-donkey-anti-chicken-secondary-antibody
RRID AB_1850023

Validation

Antibodies were validated by their suppliers and are listed in Supplementary Table 2. Information on validation from suppliers is presented below. More information on validation can be found using the Research Resource Identifier (RRID) numbers for selected antibodies.

1.
Rabbit anti-Smad3 ((phospho S423+425)[EP823Y] PUR)
Abcam
https://www.abcam.com/products/primary-antibodies/smad3-phospho-s423--s425-antibody-ep823y-ab52903.html
Research Resource Identifier (RRID) AB_882596
Host species: Rabbit
Specificity: This antibody detects Smad3 phosphorylated on Serine 423 and Serine 425. This Smad3 antibody may also detect Smad1, Smad2 and Smad5 phosphorylated at the equivalent sites.
Suitable for: WB, ICC/IF, ChIC/CUT&RUN-seq, IHC-P, Dot blot
Unsuitable for: Flow Cyt or IP
Reacts with: Mouse, Human
Predicted to work with: Drosophila melanogaster
Immunogen: Synthetic peptide. This information is proprietary to Abcam and/or its suppliers.
Positive control. WB: HL-60 treated with TGF-ß cell lysates; A549 untreated and treated with 5ng/ml TGF-ß1 for 24 hours whole cell lysates; F9 whole cell lysate. IHC-P: Human stomach and liver carcinoma tissue; Mouse kidney tissue; Environmental enteropathy (EE) duodenal biopsy. ICC/IF: TGFß treated A549 cells; PML+/+ mouse embryonic fibroblasts (MEFs) were transfected with either CTL-siRNAs or NDRG1-siRNAs; Mouse primary embryonic epicardial cells.

2.
Donkey anti-rabbit IgG Alexa Fluor 647
Invitrogen/Thermo Fisher Scientific
https://www.thermofisher.com/antibody/product/Donkey-anti-Rabbit-IgG-H-L-Highly-Cross-Adsorbed-Secondary-Antibody-Polyclonal/A-31573
RRID AB_2536183
To minimize cross-reactivity, these donkey anti-rabbit IgG whole antibodies have been affinity-purified and show a published cross-reactivity to rat IgG. Cross-adsorption or pre-adsorption is a purification step to increase specificity of the antibody resulting in higher sensitivity and less background staining. The secondary antibody solution is passed through a column matrix containing immobilized serum proteins from potentially cross-reactive species. Only the nonspecific-binding secondary antibodies are captured in the column, and the highly specific secondaries flow through. The benefits of this extra step are apparent in multiplexing/multicolor-staining experiments (e.g., flow cytometry) where there is potential cross-reactivity with other primary antibodies or in tissue/cell fluorescent staining experiments where there may be the presence of endogenous immunoglobulins.
Alexa Fluor dyes are among the most trusted fluorescent dyes available today. Invitrogen™ Alexa Fluor 647 dye is a near-infrared-fluorescent dye with excitation ideally suited to the 647 nm laser line. For stable signal generation in imaging and flow cytometry, Alexa Fluor 647 dye is pH-insensitive over a wide molar range. Probes with high fluorescence quantum yield and high photostability allow detection of low-abundance biological structures with great sensitivity. Alexa Fluor 647 dye molecules can be attached to proteins at high molar ratios without significant self-quenching, enabling brighter conjugates and more sensitive detection. The degree of labeling for each conjugate is typically 2-8 fluorophore molecules per IgG molecule; the exact degree of labeling is indicated on the certificate of analysis for each product lot.

3.
ALPL Recombinant Rabbit Monoclonal Antibody (7H11L3)
Invitrogen/Thermo Fisher Scientific
https://www.thermofisher.com/antibody/product/ALPL-Antibody-clone-7H11L3-Recombinant-Monoclonal/702454
Product Specific Information
This antibody is predicted to react with Monkey, Cat, Bovine, Rat
Recombinant rabbit monoclonal antibodies are produced using in vitro expression systems. The expression systems are developed by cloning in the specific antibody DNA sequences from immunoreactive rabbits. Then, individual clones are screened to select the best candidates for production. The advantages of using recombinant rabbit monoclonal antibodies include: better specificity and sensitivity, lot-to-lot consistency, animal origin-free formulations, and broader immunoreactivity to diverse targets due to larger rabbit immune repertoire.
Target Information
Alkaline phosphatase is a homodimeric protein enzyme (86 kDa), containing two zinc atoms crucial to its catalytic function per monomer, and it is optimally active at alkaline pH environments. The enzyme is found in prokaryotes and eukaryotes, with the same general function but in different structural forms suitable to organism's environment. ALP plays a role in liver metabolism and development within the skeleton. In bacteria, ALP acts as a source of inorganic phosphate. In yeast, ALP can be a useful protein for studying membrane protein sorting.

RRID AB_2722857

4.
Mouse anti-MHC IV Alexa Fluor 488
Invitrogen/Thermo Fisher Scientific
https://tfcom-global-nginx.commerceprod.thermofisher.com/antibody/product/Myosin-4-Antibody-clone-MF20-Monoclonal/53-6503-82
Product Specific Information
Description: This MF20 monoclonal antibody recognizes the heavy chain of myosin II, specificially the light meromyosin portion, in cardiac and skeletal muscle of vertebrates. Myosin II is composed of two heavy chains and four light chains. The 220-kDa myosin heavy chain exists as four different isoforms due to alternative splicing. Myosins interact with actin and hydrolyze ATP to function in muscle contraction, cytokinesis, and phagocytosis.
The MF20 has been shown to react to myosin from a variety of mammalian, avian and amphibian species, including rat, mouse, human, chicken, zebrafish, and dog.
Applications Reported: This MF20 antibody has been reported for use in immunohistochemical staining, immunocytochemistry, and immunohistochemical staining of frozen tissue sections.
Applications Tested: This MF20 antibody has been tested by immunocytochemistry on fixed and permeabilized C2C12 cells that were differentiated for 2 days in low serum medium prior to staining. Staining can be performed using less than or equal to 5 µg/mL. It is recommended that the antibody be carefully titrated for optimal performance in the assay of interest.
Excitation: 488 nm; Emission: 519 nm; Laser: Blue Laser.
Target Information
Target protein includes a fast myosin isoform expressed in skeletal muscle and has function involved in Muscle contraction.
RRID AB_10671272

5.
Sheep anti-digoxigenin-AP, Fab fragments, AP conjugated
Roche
https://www.sigmaaldrich.com/GB/en/product/roche/11333089001
General description
Digoxigenin is a hapten which is used in labeling of nucleic acids and in detection systems.
Probes labeled with digoxigenin has greater sensitivity equivalent to that of radioactive probes. It allows faster detection, is less hazardous and has an increased shelf life.
Specificity
The polyclonal antibody from sheep is specific to digoxigenin and digoxin and shows no cross-reactivity with other steroids, such as human estrogens and androgens.
Application
Anti-Digoxigenin has been used in DNA tethering. It has been used to attach DNA molecule to the glass surface of the flow cell.
Use Anti-Digoxigenin antibody for the detection of digoxigenin-labeled compounds using:
ELISA
Immunohistocytochemistry
In situ hybridization
Western blot
RRID AB_514496

6.
Donkey anti-Sheep IgG Alexa Fluor 488
https://www.thermofisher.com/antibody/product/Donkey-anti-Sheep-IgG-H-L-Cross-Adsorbed-Secondary-Antibody-Polyclonal/A-11015
Thermo Fisher Scientific Catalogue # A-11015
Product Specific Information
To minimize cross-reactivity, these donkey anti-sheep IgG (H+L) whole secondary antibodies have been affinity purified and cross-adsorbed against mouse, rabbit, bovine, and human sera, and human IgG. Cross-adsorption or pre-adsorption is a purification step to increase specificity of the antibody resulting in higher sensitivity and less background staining. The secondary antibody solution is passed through a column matrix containing immobilized serum proteins from potentially cross-reactive species. Only the nonspecific-binding secondary antibodies are captured in the column, and the highly specific secondaries flow through. The benefits of this extra step are apparent in multiplexing/multicolor-staining experiments (e.g., flow cytometry) where there is potential cross-reactivity with other primary antibodies or in tissue/cell fluorescent staining experiments where there are may be the presence of endogenous immunoglobulins.
Alexa Fluor dyes are among the most trusted fluorescent dyes available today. Invitrogen™ Alexa Fluor 488 dye is a bright, green-fluorescent dye with excitation ideally suited to the 488 nm laser line. For stable signal generation in imaging and flow cytometry, Alexa Fluor 488 dye is pH-insensitive over a wide molar range. Probes with high fluorescence quantum yield and high photostability allow detection of low-abundance biological structures with great sensitivity. Alexa Fluor 488 dye molecules can be attached to proteins at high molar ratios without significant self-quenching, enabling brighter conjugates and more sensitive detection. The degree of labeling for each conjugate is typically 2-8 fluorophore molecules per IgG molecule; the exact degree of labeling is indicated on the certificate of analysis for each product lot.
Target Information
Anti-Sheep secondary antibodies are affinity-purified antibodies with well-characterized specificity for ovine (sheep) immunoglobulins and are useful in the detection, sorting or purification of its specified target. Secondary antibodies offer increased versatility enabling users to use many detection systems (e.g. HRP, AP, fluorescence). They can also provide greater sensitivity through signal amplification as multiple secondary antibodies can bind to a single primary antibody. Most commonly, secondary antibodies are generated by immunizing the host animal with a pooled population of immunoglobulins from the target species and

can be further purified and modified (i.e. immunoaffinity chromatography, antibody fragmentation, label conjugation, etc.) to generate highly specific reagents.

7.
Rabbit anti-His
Cell Signaling Technologyy
https://www.cellsignal.com/products/primary-antibodies/his-tag-d3i1o-xp-rabbit-mab/12698
Specificity/Sensitivity
His-Tag (D3I1O) XP® Rabbit mAb recognizes recombinant proteins containing the 6xHis epitope tag. The antibody recognizes the 6xHis-tag fused to either the amino or carboxy terminus of targeted proteins in transfected cells.
Species Reactivity:
All Species Expected
Source/Purification
Monoclonal antibody is produced by immunizing animals with a synthetic peptide corresponding to residues of the 6xHis epitope tag.
Catalogue number 12698S

8.
Mouse anti-Myc
Millipore
https://www.merckmillipore.com/GB/en/product/Anti-Myc-Tag-Antibody-clone-4A6,MM_NF-05-724
Anti-Myc Tag, clone 4A6, is a mouse monoclonal antibody that is validated for use in ChIP, IC, IF, IP, and WB for the detection of Myc Tag and does not show the very high context sensitivity.
Clone 4A6 is an antibody that recognizes ten amino acid sequence EQKLISEEDL (Glu-Gln-Lys-Leu-Iso-Ser-Glu-Glu-Asp-Leu) corresponding to the C-terminal region of human c-Myc protein (UniProt: P01106). Unlike other clones whose performance may be affected when Myc tag sequence is followed by smaller neutral amino acids, Clone 4A6 offers much more consistent detection of Myc-tagged proteins irrespective of any changes in neighboring amino acid sequences.
RRID AB_11211891

9.
Chicken anti-HA
Abcam
https://www.abcam.com/products/primary-antibodies/ha-tag-antibody-ab9111.html
Description: Chicken polyclonal to HA tag
Host species: Chicken
Specificity. ELISA: this antibody diluted at 1:20,000 gave an O.D.= 1.0 in a 15 minute reaction against peptide conjugated to a different carrier than used for anti peptide purification. HRP conjugated goat anti chicken IgG (IgY) was used and TMB was the substrate.
Tested applications. Suitable for: WB, ELISA, ICC/IF
Species reactivity. Reacts with: Species independent
Immunogen: Synthetic peptide corresponding to Influenza A HA tag conjugated to keyhole limpet haemocyanin. Influenza hemagglutinin-HA (epitope)
Positive control. ICC/IF: CHO cells transfected with 12 tags constructs (CHO-12 tags) and transfected with GFP-HA constructs (CHO-GFP-HA).
RRID AB_307020

10.
Donkey anti-Rabbit IgG IRDye 680RD
LI-COR
https://www.licor.com/bio/reagents/irdye-680rd-donkey-anti-rabbit-igg-secondary-antibody
Immunogen: Rabbit IgG
Purity and Specificity. The antibody was isolated by affinity chromatography using antigens coupled to agarose beads. Based on ELISA, this antibody reacts with the heavy and light chains of rabbit IgG, and with the light chains common to most rabbit immunoglobulins. This antibody was tested by ELISA and/or solid-phase adsorbed to ensure minimal cross-reactivity with bovine, chicken, goat, guinea pig, hamster, horse, human, mouse, rat, and sheep serum proteins, but may cross-react with immunoglobulins from other species. The conjugate has been specifically tested and qualified for Western blot and In-Cell Western Assay applications.
Applications. Highly recommended for:
Western Blot
In-Cell Western Assay
On-Cell Western Assay
Protein Array
Immunohistochemistry
Small Animal Imaging
Microscopy
2D Gel Detection
Tissue Section Imaging
RRID AB_2716687

11.
Donkey anti-Mouse IgG IRDye 800CW
LI-COR

https://www.licor.com/bio/reagents/irdye-800cw-donkey-anti-mouse-igg-secondary-antibody
Immunogen: Mouse IgG
Purity and Specificity. The antibody was isolated by affinity chromatography using antigens coupled to agarose beads. Based on ELISA, this antibody reacts with the heavy and light chains of mouse IgG, and with the light chains of mouse IgM and IgA. This antibody was tested by ELISA and/or solid-phase adsorbed to ensure minimal cross-reactivity with bovine, chicken, goat, guinea pig, horse, human, rabbit, and sheep serum proteins, but may cross-react with immunoglobulins from other species. The conjugate has been specifically tested and qualified for Western blot and In-Cell Western Assay applications.
Applications. Highly Recommended for:
Western Blot
In-Cell Western Assay
On-Cell Western Assay
Protein Array
Immunohistochemistry
Small Animal Imaging
Microscopy
2D Gel Detection
Tissue Section Imaging
Virus Titration Assay
RRID AB_621847

12.
Donkey anti-Chicken IgG IRDye 800CW
https://www.licor.com/bio/reagents/irdye-800cw-donkey-anti-chicken-secondary-antibody
Immunogen: Chicken IgY, whole molecule. IgY is the original designation for the IgG-like protein found in both serum and egg yolk.
Purity and Specificity. The antibody was isolated from antisera by immunoaffinity chromatography using antigens coupled to agarose beads. Based on immunoelectrophoresis and/or ELISA, this antibody reacts with whole molecule chicken IgY, and with the light chains of other chicken immunoglobulins. No reactivity was detected against non-immunoglobulin serum proteins. This antibody was tested by ELISA and/or solid-phase adsorbed to ensure minimal cross-reactivity with bovine, goat, guinea pig, Syrian hamster, horse, human, mouse, rabbit, rat, and sheep serum proteins, but may cross-react with immunoglobulins from other species. The conjugate has been specifically tested and qualified for Western blot applications.
Applications. Highly recommended for:
Western Blot
In-Cell Western Assay
On-Cell Western Assay
Protein Array
Immunohistochemistry
Small Animal Imaging
Microscopy
2D Gel Detection
Tissue Section Imaging
Virus Titration Assay
RRID AB_1850023

Eukaryotic cell lines

Policy information about cell lines and Sex and Gender in Research

Cell line source(s)	C2C12 immortalized mouse myoblasts stably transfected with a reporter plasmid of BMP signaling (Herrera B, Inman GJ. A rapid and sensitive bioassay for the simultaneous measurement of multiple bone morphogenetic proteins. Identification and quantification of BMP4, BMP6 and BMP9 in bovine and human serum. BMC Cell Biol 10, 20 (2009)).<br><br>Human embryonic kidney (HEK) 293T cells from ATCC (Catalogue number CRL-11268).<br><br>Drosophila S2R+ (Sawala A, Sutcliffe C, Ashe HL. Multistep molecular mechanism for bone morphogenetic protein extracellular transport in the Drosophila embryo. Proc Natl Acad Sci U S A 109, 11222-11227 (2012)).
Authentication	Cell lines were not experimentally authenticated for these studies.
Mycoplasma contamination	HEK293T and C2C12 cell lines were tested for mycoplasma contamination in Prof. Christian Siebold's lab by Dr. Rebekka Siebold-Schwab, and were negative for mycoplasma contamination. Other cell lines were not tested for mycoplasma contamination.
Commonly misidentified lines (See ICLAC register)	No commonly misidentified cell lines were used.

Animals and other research organisms

Policy information about studies involving animals; ARRIVE guidelines recommended for reporting animal research, and Sex and Gender in Research

Laboratory animals	Drosophila melanogaster. Adult males and females. Embryos were fixed 2-4 hours after egg lay. Details of lines generated in this work can be found in the Methods section and details of other lines used can be found at the Bloomington Drosophila Stock Centre using identifier numbers provided in Supplementary Table 2.<br><br>Mus musculus. All procedures were carried out in accordance with UK Home Office regulations and the Animals (Scientific Procedures) Act 1986. All mice were housed in individually ventilated cages at the animal unit at the Functional Genetics Facility (Wellcome Centre for Human Genetics, University of Oxford). They were housed in a specific-pathogen-free (SPF) facility with unrestricted access to food and water and had not been involved in any previous procedures. All strains were maintained on a C57BL/6J background for R6 generations. Procedures were conducted on mice at least 6 weeks of age, including both males and females.
Wild animals	This study did not involve wild animals.
Reporting on sex	Sex was not considered in this study.
Field-collected samples	This study did not involve field-collected samples.
Ethics oversight	No ethical approval or guidance was required for Drosophila studies. Invertebrate animals such as Drosophila are not protected by the law or monitored by the UK Home Office.<br><br>All procedures on mice were carried out in accordance with UK Home Office regulations and the Animals (Scientific Procedures) Act 1986.

Note that full information on the approval of the study protocol must also be provided in the manuscript.

Plants

Seed stocks	N/A
Novel plant genotypes	N/A
Authentication	N/A
